# Isolation of adipose tissue derived regenerative cells from human subcutaneous tissue with or without the use of an enzymatic reagent

**DOI:** 10.1371/journal.pone.0221457

**Published:** 2019-09-03

**Authors:** Glenn E. Winnier, Nick Valenzuela, Jennifer Peters-Hall, Joshua Kellner, Christopher Alt, Eckhard U. Alt

**Affiliations:** 1 InGeneron, Inc., Houston, TX, United States of America; 2 Heart and Vascular Institute, Department of Medicine, Tulane University Health Science Center, New Orleans, LA, United States of America; 3 Sanford Health, University of South Dakota, Sioux Falls, SD, United States of America; 4 Isar Klinikum Munich, Munich, Germany; Augusta University, UNITED STATES

## Abstract

Freshly isolated, uncultured, autologous adipose derived regenerative cells (ADRCs) have emerged as a promising tool for regenerative cell therapy. The Transpose RT system (InGeneron, Inc., Houston, TX, USA) is a system for isolating ADRCs from adipose tissue, commercially available in Europe as a CE-marked medical device and under clinical evaluation in the United States. This system makes use of the proprietary, enzymatic Matrase Reagent for isolating cells. The present study addressed the question whether the use of Matrase Reagent influences cell yield, cell viability, live cell yield, biological characteristics, physiological functions or structural properties of the ADRCs in final cell suspension. Identical samples of subcutaneous adipose tissue from 12 subjects undergoing elective lipoplasty were processed either with or without the use of Matrase Reagent. Then, characteristics of the ADRCs in the respective final cell suspensions were evaluated. Compared to non-enzymatic isolation, enzymatic isolation resulted in approximately twelve times higher mean cell yield (i.e., numbers of viable cells/ml lipoaspirate) and approximately 16 times more colony forming units. Despite these differences, cells isolated from lipoaspirate both with and without the use of Matrase Reagent were independently able to differentiate into cells of all three germ layers. This indicates that biological characteristics, physiological functions or structural properties relevant for the intended use were not altered or induced using Matrase Reagent. A comprehensive literature review demonstrated that isolation of ADRCs from lipoaspirate using the Transpose RT system and the Matrase Reagent results in the highest viable cell yield among published data regarding isolation of ADRCs from lipoaspirate.

## Introduction

Regenerative cell therapy, which refers to the therapeutic application of stem cells to repair diseased or injured tissue, has received increasing attention from basic scientists, clinicians and the public (e.g., [1–5]). Stem cells hold significant promise for tissue regeneration due to their innate ability to provide a renewable supply of progenitor cells that can form multiple cell types, whole tissue structures, and even organs (e.g., [1–5]).

Over the last years, freshly isolated, uncultured, autologous adipose-derived regenerative cells (UA-ADRCs) have become highly attractive for the practice of regenerative cell therapy (e.g., [6–10]) (note that freshly isolated, uncultured, adipose-derived regenerative cells were also named “stromal vascular fraction” (SVF) in many publications; e.g., [11–13]). This is due to the fact that UA-ADRCs have several advantages over other types of cells used in and/or under investigation for regenerative cell therapy. First of all, UA-ADRCs do not share ethical concerns nor the risk of teratoma formation reported for embryonic stem cells [14–16]. Neither do UA-ADRCs share the risk of tumorigenesis that severely limits the clinical translation of induced pluripotent stem (iPS) cells [17–19]. Second, because UA-ADRCs are autologous cells, their application does not bear the risk of HLA mismatch associated with allogeneic cells (compromised clinical outcome after application of allogeneic cells was reported in [20–22]). Third, adipose tissue typically has a significantly higher stem cell density than bone marrow (5 to 10% vs. 0.1%), and harvesting adipose tissue can be less invasive than harvesting bone marrow [23, 24]. Fourth, unlike the use of adipose derived stem cells (ASCs) which are culture-expanded from the SVF [25–27], the use of UA-ADRCs allows immediate usage at point of care [8–10]. This is combined with low safety concerns as no culturing or modification are applied. Of note, when using UA-ADRCs during a surgical procedure in an autologous and homologous way, they are not considered an advanced therapy medicinal product (ATMP) by the European Medicines Agency [28]. In contrast, expansion of ASCs in vitro may be associated with risks such as possible loss of stemness or cell transformation [29–31]. On the other hand, recent studies on culture systems and animal models indicated non-inferiority or even superiority of UA-ADRCs over ASCs in, for example, tendon healing [32], bone regeneration [33] and rescuing heart function after acute myocardial infarction [34] (see also [13]).

For obvious reasons, an optimal system for providing UA-ADRCs at point of care should be capable of isolating the highest possible number of living ADRCs from the lowest possible amount of adipose tissue in the shortest possible time, and providing the cells at the highest possible concentration in a final cell suspension.

**Fig 1:**
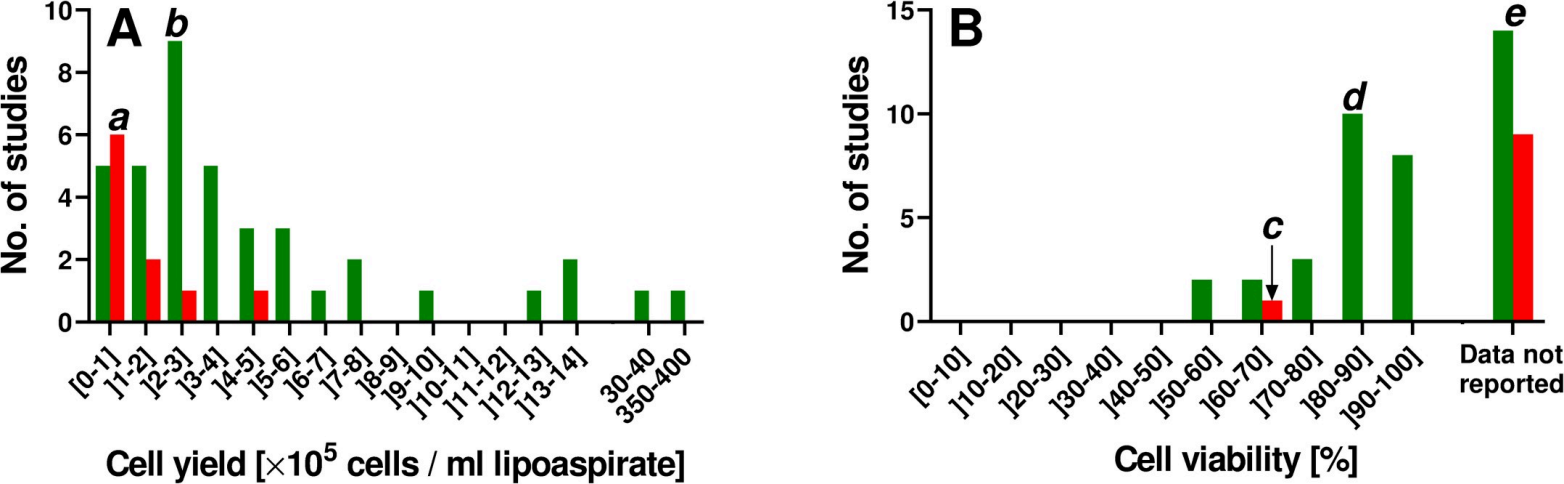
**Frequency distributions of cell yield (A) and cell viability (B) data reported in the literature for enzymatic (green) and non-enzymatic (red) methods for isolating ADRCs from adipose tissue.** Interpretation of this data is illustrated by the following examples: (i) For six out of ten non-enzymatic methods (60%) cell yield between 0 and 1×10^5^ cells per ml lipoaspirate was reported (“*a*” in Panel A). (ii) For nine out of 39 enzymatic methods (21%) cell yield between 2×10^5^ and 3×10^5^ cells per ml lipoaspirate was reported (“*b*” in Panel A). (iii) For only one out of ten non-enzymatic methods (10%) cell viability was reported (between 60.1% and 70%) (“*c*” in Panel B). (iv) For ten out of 39 enzymatic methods (20.5%) cell viability between 80.1% and 90% was reported (“*d*” in Panel B). (v) Unfortunately, for nine out of ten non-enzymatic methods (90%) as well as for 14 out of 39 enzymatic methods (36%) no cell viability data were reported (“*e*” in Panel B).

A number of enzymatic and non-enzymatic methods were reported for isolating human ADRCs (Tables 1 and 2; c.f. also Fig 1 and [35–38]). For enzymatic methods, cell yield between 0.0 [39] and 387×10^5^ cells per ml lipoaspirate [40] were reported (Table 1) (mean, 14.6×10^5^; standard deviation, 61.5×10^5^; median, 3.1×10^5^; for 90% of the methods listed in Table 1 cell yield between 0.0 and 12.2×10^5^ cells per ml lipoaspirate was reported; Fig 1). Related cell viability data (i.e., relative number of living cells) varied between 50% [41] and 94% [23] (note that for 14 out of the 39 enzymatic methods no cell viability data were reported; Table 1).

**Table 1:** Cell yield, cell viability, number of living cells per gram lipoaspirate and other details reported in studies describing enzymatic methods for isolating ADRCs.

R	Y	S	O/C	M/S/A	CY	V	LCY	V_L_	PT	FV
[39]	2015	(a)	O	M	**0.0**	--	--	80	--	--
[41]	2013	(b)	C	S	**0.07**	87	0.06	130	88	--
[41]	2013	(c)	C	M	**0.35**	72	0.25	80	111	--
[44]	2014	(d)	C	A	**0.72**	91	0.66	250	98	--
[45]	2015		C	A	**1.00**	>90	0.90	500	133	11
[46]	2016	(e)	C	A	**1.01**	84	0.85	162	89	5
[45]	2015		O	M	**1.03**	>90	0.92	--	--	--
[41]	2013	(f)	O	M	**1.07**	57	0.61	125	115	--
[45]	2015		O	M	**1.47**	--	--	212	--	--
[47]	2012		O	M	**1.60**	>90	1.44	220	--	--
[48]	2014		O	M	**2.30**	--	--	--	--	--
[41]	2013	(g)	C	A	**2.41**	93	2.24	140	90	--
[39]	2015	(h)	C	A	**2.55**	--	--	80	--	--
[47]	2012		C	S	**2.60**	>90	2.34	220	--	10
[46]	2016	(i)	C	M	**2.85**	69	1.96	102	71	7
[49]	2019		O	M	**2.93**	76	2.23	100	82	--
[50]	2015	(j)	C	A	**2.79**	88	2.46	181	120	8
[51]	2008	(k)	C	A	**2.95**	87	2.57	86	--	--
[52]	2005	(l)	C	A	**3.00**	93	2.79	--	--	--
[53]	2006		O	M	**3.08**	--	--	--	--	--
[54]	2014	(m)	C	A	**3.60**	85	3.06	--	--	5
[55]	2014	(n)	C	M	**3.60**	83	2.98	400	--	--
[43]	2014		O	M	**3.60**	67	2.41	--	--	--
[56]	2014		O	M	**3.68**	75	2.76	--	--	--
[23]	2004		O	M	**4.04**	94	3.80	--	--	--
[57]	2013		O	M	**4.07**	--	--	24	--	--
[58]	2013		O	M	**4.80**	--	--	55	--	--
[42]	2015	(o)	C	M	**5.34**	--	--	--	--	--
[49]	2019		O	M	**5.35**	86	4.60	100	72	--
[46]	2016	(p)	O	M	**5.36**	82	4.39	156	65	12
[46]	2016	(q)	C	M	**6.25**	50	3.12	140	121	20
[59]	2013		O	M	**7.01**	82	5.75	50	--	--
[59]	2013		C	A	**7.02**	81	5.69	50	--	--
[60]	2017	(r)	C	M	**9.06**	--	--	50	120	--
[42]	2015	(s)	C	A	**12.1**	--	--	--	--	--
[61]	2006		O	M	**13.1**	--	--	--	--	--
[62]	2001		O	M	**13.3**	--	--	300	--	--
[63]	2016	(t)	C	M	**35.0**	--	--		80	5
[40]	2016		O	M	**387**	--	--	80	--	--

Abbreviations: R, reference; Y, year of publication; S, name of commercial system (if applicable; information is given here as provided in the corresponding references); O, open isolation system; C, closed isolation system; M, manual method; S, semiautomated method; A, automated method; CY, cell yield [×10^5^/ml lipoaspirate]; CV, cell viability [%]; LCY, live cell yield (i.e., number of living cells) [×10^5^/ml lipoaspirate]; V_L_, average lipoaspirate volume that was processed [ml]; PT, average processing time [min]; FY, volume of the final cell suspension; --, data not provided or could not be calculated (in case of NLC).

(a), Medikhan centrifuge (Medi-Khan Inc., West Hollywood, CA, USA)

(b), Cha-Station (CHA Biotech, Kangnamgu, Republic of Korea)

(c), Lipokit with MaxStem (Medi-Khan Inc., West Hollywood, CA, USA)

(d), Celution System (Cytori Therapeutics, San Diego, CA, USA)

(e), Cytori StemSource 900/MB System (Cytori Therapeutics)

(f), Multi Station (PNC International, Gyeonggido, Republic of Korea)

(g), Celution800/CRS System (Cytori Therapeutics)

(h), Celution System (Cytori Therapeutics)

(i), GID SVF-2 platform (The GID Group, Inc., Louisville, CO, USA)

(j), Celution800/CRS System (Cytori Therapeutics)

(k), Celution System (Cytori Therapeutics)

(l), Celution800/CRS System (Cytori Therapeutics)

(m), Celution System (Cytori Therapeutics)

(n), GID SVF-1 device (The GID Group, Inc., Louisville, CO, USA)

(o), Lipokit Medikhan System (Medikan International Inc., Pusan, Korea)

(p), MultiStation (PNC International, Gyeonggido, Republic of Korea)

(q), LipoKit platform (Medi-Khan, Inc., Irwindale, CA, USA)

(r), Lipokit 416D (Medikhan, Seocho-gu, Seoul, South Korea)

(s), Celution System (Cytori Therapeutics)

(t), centrifuge (Lipokit; Medikhan) and shaker/incubator (Celltibator; Medikhan).

**Table 2:** Cell yield, cell viability, number of living cells per gram lipoaspirate and other details reported in studies describing non-enzymatic methods for isolating ADRCs.

R	Y	S	O/C	M/S/A	CY	CV	LCY	V_L_	PT	FV
[39]	2015	(u)	O	S	**0.07**	--	--	80	--	--
[43]	2014		O	M	**0.11**	69	0.08	--	--	--
[48]	2014		O	M	**0.12**	--	--	--	--	--
[48]	2014		O	M	**0.23**	--	--	--	--	--
[58]	2013		O	M	**0.25**	--	--	180	--	--
[39]	2015	(v)	O	M	**0.30**	--	--	80	--	--
[64]	2014		O	M	**1.25**	--	--	80	15	--
[56]	2014	(i)	C	S	**1.39**	--	--	200	--	--
[65]	2009		O	M	**2.40**	--	--	--	--	--
[42]	2015	(j)			**4.44**	--	--	80	--	--

Abbreviations: R, reference; Y, year of publication; S, name of commercial system (if applicable; information is given here as provided in the corresponding references); O, open isolation system; C, closed isolation system; M, manual method; S, semiautomated method; A, automated method; CY, cell yield [×10^5^/ml lipoaspirate]; CV, cell viability [%]; LCY, live cell yield (i.e., number of living cells) [×10^5^/ml lipoaspirate]; V_L_, average lipoaspirate volume that was processed [ml]; PT, average processing time [min]; FY, volume of the final cell suspension; --, data not provided or could not be calculated (in case of NLC).

(u), Mystem kit (Bi-Medica, Treviolo, Italy)

(v), Fatstem (CORIOS Soc. Coop, San Giuliano Milanese, Italy)

(w), StromaCell mechanical cell-separation device (MicroAire Aesthetics, Charlottesville, VA, USA)

(x), Fastem Corios (CORIOS Soc. Coop, San Giuliano Milanese, Italy).

For non-enzymatic methods, cell yield between 0.07×10^5^ [39] and 4.44×10^5^ [42] cells per ml lipoaspirate were reported (Table 2) (mean, 1.1×10^5^; standard deviation, 1.4×10^5^; median, 0.3×10^5^; for 90% of the methods listed in Table 2 cell yield between 0.07×10^5^ and 2.4×10^5^ cells per ml lipoaspirate was reported; Fig 1). Of note, except for one method [43] no cell viability data were reported for non-enzymatic methods (Table 2).

Altogether it follows from these data that (i) both non-enzymatic and enzymatic methods for isolating ADRCs from adipose tissue can result in significant variability in cell yield (Fig 1A); (ii) cell viability data were reported for the majority of enzymatic methods for isolating ADRCs from adipose tissue (25 out of 39 methods; 64%), with cell viability of 80% or higher reported for 18 out of 25 methods (72%) (Fig 1B); (iii) the data reported so far do not allow conclusions regarding cell viability of ADRCs isolated from adipose tissue with non-enzymatic methods (Fig 1B); and (vi) enzymatic isolation of ADRCs from adipose tissue generally results in higher cell yield than non-enzymatic isolation (Fig 1A).

The present study tested the following hypotheses: (i) Isolation of ADRCs from human adipose tissue with the Transpose RT system and the proprietary enzymatic Matrase Reagent (both commercially available from InGeneron, Inc., Houston, TX, USA) (thereafter: “Transpose RT / Matrase isolation” and “Transpose RT / Matrase isolated ADRCs”) results in significantly higher cell yield, cell viability and number of living cells per ml lipoaspirate than isolation of ADRCs from human adipose tissue without the use of Matrase Reagent but under otherwise identical processing conditions (thereafter: “Transpose RT / no Matrase isolation” and “Transpose RT / no Matrase isolated ADRCs”), but does not influence biological characteristics, physiological functions or structural properties of the ADRCs in the final cell suspension. (ii) Transpose RT / Matrase isolation of ADRCs from human adipose tissue results in a higher live cell yield than the methods reported in those studies that are listed in Tables 1 and 2.

## Materials and methods

### Isolation of cells from subcutaneous adipose tissue

Subcutaneous adipose tissue was recovered by a medical practitioner from subjects via lipoaspiration undergoing elective lipoplasty according to standard procedures with informed consent. N = 12 subjects with age ranging between 32 and 59 years (Table 3) (and three additional subjects for testing of residual collagenase activity in the final cell suspension) were consented according to the IntegReview IRB approved protocol #200601001 (IntegReview IRB, Austin, TX, USA).

**Table 3:** Subjects’ demographics.

Subject #	Age	Sex	Race
1	36	Female	Caucasian
2	59	Female	Caucasian
3	46	Female	Caucasian
4	49	Female	Caucasian
5	37	Female	Caucasian
6	36	Female	Caucasian
7	39	Female	Black
8	36	Female	Caucasian
9	32	Female	Hispanic
10	32	Female	Hispanic
11	38	Female	Caucasian
12	42	Female	Caucasian

A sample of recovered lipoaspirate from each subject was divided into two equal parts of 25 ml each, and was processed either with the use of Matrase Reagent (InGeneron) (Transpose RT / Matrase isolation) or only mechanically without the use of Matrase Reagent but under otherwise identical processing conditions (Transpose RT / no Matrase isolation). The Matrase Reagent is a GMP certified, proprietary enzyme blend of collagenase and neutral protease.

**Fig 2:**
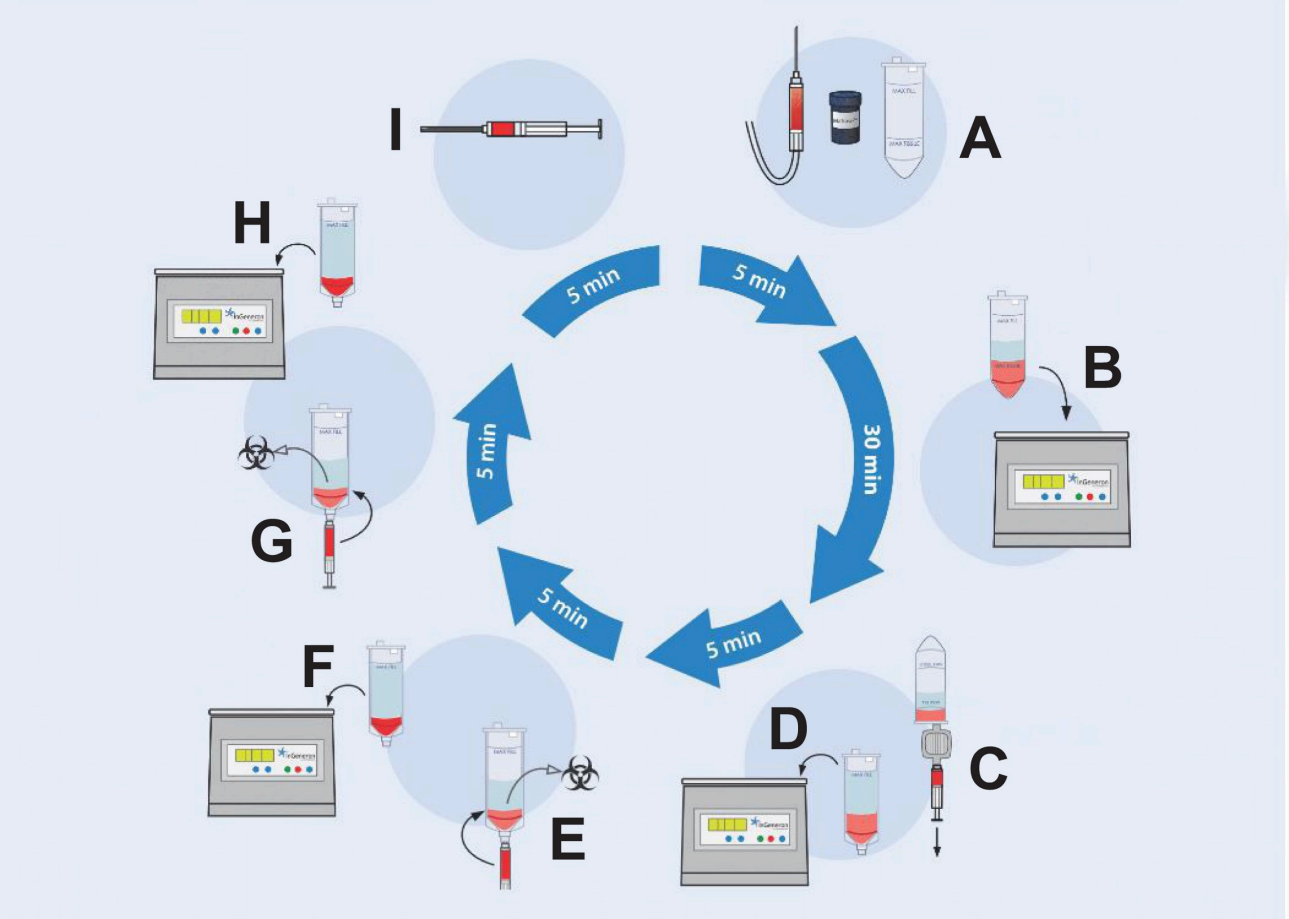
Isolation of ADRCs from human adipose tissue with the Transpose RT system and the Matrase Reagent (both from InGeneron, Inc., Houston, TX, USA). Details are provided in the text.

Processing was performed as described in the tissue processing procedure section found in the 11011E Transpose RT Instructions for Use (11011–01 IFU; InGeneron, Inc.) (Fig 2), comprising the following steps: (i) The recovered lipoaspirate (25 ml) was loaded together with 2.5 ml reconstituted Matrase (in case of Transpose RT / Matrase isolation) and lactated Ringer solution (preheated to 39° C) into a processing tube up to the MAX FILL line (Fig 2A). (ii) The filled processing tubes were subjected in an inverted position inside the Transpose RT system to repetitive acceleration and deceleration for 30 minutes at 39° C (Fig 2B). (iii) The processed lipoaspirate solution was filtered through a 200 μm filter (Fig 2C) and transferred into a wash tube. After filling the wash tube with saline (room temperature) up to the MAX FILL line, the cells were separated from the rest of the tissue by centrifugation at 600g for 5 minutes at room temperature (Fig 2D). Then, the SVF/ ADRCs (approximately 2 ml) were extracted through a swabable luer vial adapter at the bottom of the wash tube, and the remaining substances (fat, debris and liquid) were discarded (Fig 2E). (v) The cells were returned into the empty wash tube and (after adding fresh saline up to the MAX FILL line) centrifugated again for 5 minutes (Fig 2F). (vi) The previous washing step was repeated (Fig 2G and 2H). (vii) Finally, the concentrated SVF/ ADRCs (approximately 3 ml) were extracted (Fig 2I) and slowly pushed through a luer coupler into a new sterile syringe for further application to the patient.

The final cell suspension was analyzed for cell yield, cell viability, number of living cells per ml lipoaspirate and cell size. The respective differentiation capacity into the three germ layers was assessed for both Transpose RT / Matrase isolated ADRCs and Transpose RT / no Matrase isolated ADRCs.

### SVF yield statistics, expansion, and cryopreservation

Cell counts and viability were determined using the FDA approved NucleoCounter NC-200 device (ChemoMetec Inc., Bohemia, NY, USA) as described by the manufacturer’s protocol.

For expansion, SVF was plated at a cell density of 7×10^6^ in T75 tissue culture flasks in 15 ml of MesenCult MSC basal medium (Stem Cell Technologies, Cambridge, MA, USA) supplemented with MesenCult MSC Stimulatory Supplement, PenStrep (Gibco, Waltham, MA, USA), Fungizone (Life Technologies, Carlsbad, CA, USA) and L-Glutamine (Corning Life Sciences, Tewksbury, MA, USA), at 37° C under 5% CO_2_. Cells were passed once reaching 75% confluency using 0.25% Trypsin (Sigma-Aldrich, St. Louis, MO, USA) for 5 min at 37° C. Then, cells were plated at a cell density of 7.5×10^5^ in T75 tissue culture flasks in 15 ml of complete MSC medium. Cells were cryopreserved by resuspending ASC cell pellets in Prime-XV MSC FreezIS DMSO-free media (Irving Scientific, Santa Ana, CA, USA) at cell densities between 2×10^6^ and 5×10^6^ cells/ml. The samples were frozen overnight at -80°C in freezing containers designed to cool at a rate of -1° C/min (Mr. Frosty, Thermo Scientific, Waltham, MA, USA).

### Analysis of colony forming units

The colony forming unit (CFU) assay was performed according to [66]. To this end, freshly isolated SVF/ADRCs from each subject were plated into a 6-well plate (Corning Life Sciences) at two different cell densities. The cells were plated at densities of 50,000 and 100,000 total cells in 2 ml of complete MSC medium in each well; these experiments were repeated in triplicate. Cells were incubated at 37° C under 5% CO_2_ for 14 days to allow CFUs to form. Medium was changed every 2–4 days. After day 14 the medium was aspirated, the cells were washed twice in PBS and fixed in 2 ml of 10% formalin at room temperature for 30 min with gentle rocking. Cells were then washed three times with DI water and stained with 2 ml hematoxylin (Bio-Rad Laboratories, Hercules, CA, USA) for 15 min at room temperature. The cells were then washed with warm tap water until the wash ran clear. CFUs were quantified by manually counting the entire well; each sample was counted at both cell concentrations and in triplicate. Colonies containing > 50 fibroblast colony-forming units (CFU-F) were counted. CFU-F frequency was calculated by dividing the number of colonies by the number of seeded cells.

### Analysis of embryoid body formation

Embryoid bodies are defined as spherical clusters of both pluripotent and committed stem cells that can organize in a developmental-specific manner and give rise to mature cells from any differentiation lineage (reviewed in, e.g., [67].

To form embryoid bodies, freshly isolated ADRCs were plated into ultra-low adherent flasks (Corning Life Sciences) at a density of 60,000 cells/cm^2^ in GMEM (Gibco) supplemented with 2 mM L-Glutamine (Corning Life Sciences), non-essential amino acids (Sigma-Aldrich), B27 (Life Technologies), 0.6% glucose (Sigma-Aldrich), 10 ng/ml human bFGF (Invitrogen Life Technologies), 20 ng/ml human EGF (Life Technologies), 1 U/ml human thrombin (EMD Millipore, Burlington, MA, USA) and 2 μg/ml ciprofloxacin (Sigma-Aldrich). Cells were incubated at 37° C under 5% CO_2_ and half of the medium was changed every 3 days. The diameter of embryoid bodies was measured on photomicrographs using ImageJ software.

### Differentiation assays

ASCs on their 3^rd^ or 6^th^ passage were assayed for differentiative potential into adipogenic, osteogenic, hepatogenic and neurogenic cell lines.

#### Adipogenic differentiation

ASCs on their 3^rd^ passage were plated onto a 12 well plate at 40,000 cells per well in 1.5 ml of complete MSC medium and allowed to grow for 2 days. On day 3 all of the medium was aspirated and replaced with either complete MSC medium or StemPro Adipogenic differentiation medium (Life Technologies) and incubated for 2 weeks, changing media every 3–4 days. Then, the presence of intracytoplasmic lipids (triglycerides) was assessed with Oil red-O staining as previously described [60]. Percentage of adipocytes was calculated by microscopic inspection. The percentage of adipocytes was determined by calculating the ratio of Oil red-O positive cells versus total cells.

##### Osteogenic differentiation

ASCs on their 3^rd^ passage were plated onto a 12 well plate at 20,000 cells per well in 1.5 ml of complete MSC medium and allowed to grow for 2 days. On day 3 all of the medium was aspirated and replaced with either complete MSC medium or StemPro Osteogenic Differentiation medium (Life Technologies) and incubated for 2 weeks, changing media every 3–4 days. Then, the presence of calcific deposits was investigated with Alizarin red staining (Alfa Aesar, Haverhill, MA, USA) as described in the protocol.

##### Hepatogenic differentiation

ASCs on their 3^rd^ passage were plated onto a 12 well plate at 20,000 cells per well in 1.5 ml of MesenCult MSC medium (Stem Cell Technologies) and allowed to grow for 2 days. Hepatogenic differentiation was achieved using the Human Mesenchymal Stem Cell Hepatogenic Differentiation Medium kit (Cyagen, Santa Clara, CA, USA). Then, the presence of structures containing a high proportion of carbohydrate macromolecules (glycogen, glycoprotein and proteoglycans) was investigated with Periodic Acid Schiff staining (Sigma-Aldrich) as described in the protocol.

##### Neurogenic differentiation

Nunc Lab-Tek II 4-well chamber slides (Thermo Fisher) were pre-coated with Poly-D-Lysine (Trevigen, Gaithersburg, MD, USA)/Laminin (Gibco) solution (10 μg/ml each) on day 0. Then, 20.000 ASCs on their 6^th^ passage were seeded per well on day 1 and cultured for 24 hours before exposure to Mesencult MSC medium (Stem Cell Technologies) or neurogenic differentiation medium. The latter consisted of Neurobasal Medium (Thermo Fisher) supplemented with 2% B27 (Thermo Fisher) and containing 10 μM Forskolin (Stem Cell Technologies), 5 μg/ml insulin (Sigma-Aldrich), 500 μM 1-methyl-3-isobutylxanthine (IBMX) (Sigma-Aldrich), 50 μM ascorbic acid (Sigma-Aldrich), 10 ng/ml nerve growth factor (NGF) (Thermo Fisher), 1% L-Glutamine (Corning Life Sciences) and 1% Pen/Strep (Gibco). Cells were cultured in neurogenic differentiation medium for 21 days with media changes every 3–4 days.

After 21 days of culturing cells in neurogenic differentiation medium or control medium, they were fixed with 10% formalin (Sigma-Aldrich). Then, cells were treated with PBS containing 0.3% Triton X-100 (Sigma-Aldrich) and 5% normal goat serum (Jackson Immunoresearch, West Grove, PA, USA) at room temperature for 1 h to block non-specific binding sites prior to the addition of primary antibodies. Afterwards, cells were incubated with primary antibodies against microtubule-associated protein 2 (MAP2) (rabbit polyclonal, 1:100, ab32454; Abcam, Cambridge, UK) or Beta III Tubulin (β3TUB) (rabbit monoclonal, 1:200, β3-Tubulin (D71G9) XP Rabbit mAb #5568; Cell Signaling Technology, Danvers, MA, USA) for 2 h at room temperature. (MAP2 is a neuron-specific cytoskeletal protein that is used as a marker of neuronal phenotype [68], and β3TUB is a tubulin thought to be specifically involved during differentiation of neuronal cell types [69].) Antibodies were diluted in antibody dilution buffer (PBS containing 1% bovine serum albumin (VWR, Radnor, PA, USA) and 0.3% Triton X-100 (Sigma-Aldrich)). After incubation with primary antibodies, the cells were washed three times with PBS, followed by incubation with goat anti-rabbit secondary antibody conjugated with Alexa Fluor 594 (Thermo Fisher) for 1 h at room temperature. Then, cells were again washed three times with PBS, followed by counterstaining with DAPI (Sigma-Aldrich). Finally, chambers were removed from the slides, and coverslips were mounted with Vectashield Antifade Mounting Medium (Vector Laboratories, Burlingame, CA, USA).

PC-12 cells (a model system for neuronal differentiation [70]) were used as positive controls. PC-12 cells were seeded onto COL IV (Santa Cruz, Santa Cruz, CA, USA) coated Nunc Lab-Tek II 4-well chamber slides, with 8,000 cells per well in neurogenic culture medium. Cells were cultured for 6 days and fixed with 10% formalin. Immunofluorescent detection of MAP2 and β3TUB was performed as described for ASCs.

### RNA isolation and quantitative PCR

RNA isolation was performed using Trizol (Life Technologies) in accordance with the manufacturer’s protocol. Total RNA was purified using the Direct-Zol RNA miniprep kit (Zymo Research, Irvine, CA, USA) as described in the protocol. cDNA was generated using iScript Reverse Transcriptase SuperMix (Bio-Rad Laboratories). Then, relative mRNA levels of Oct4 (a transcription factor associated with self-renewal [71], Klf4 (a marker of stemness [71]) and Hes1 (another known stem cell marker [72]) were measured using the SsoAdvanced Universal SYBR Green Supermix (Bio-Rad Laboratories) according to the manufacturer’s protocols in a Bio-Rad CFX96 Real-Time PCR detection system (Bio-Rad Laboratories). All samples were analyzed in triplicate. Qualification of RNA and cDNA was performed using a NanoDrop spectrometer (Thermo Fisher Scientific). Primer probe sets were custom oligos (Sigma-Adrich) (Table 4).

**Table 4:** Primer probe sets used in the present study.

Gene	Oligos
Oct4	Oct4-F: 5′-GCAAGCCCTCATTTCACCA-3’ Oct4-R: 5′-GCCCATCACCTCCACCAC-3′
Klf4	Klf4-F: 5'-AAGAGTTCCCATCTCAAGGCACA-3’ Klf4-R: 5'-GGGCGAATTTCCATCCACAG-3’
Hes1	Hes1-F: 5'-CCTGTCATCCCCGTCTACAC-3’ Hes1-R: 5'-CACATGGAGTCCGCCGTAA-3’
GAPDH*	GAPDH-F: 5'-CGCTCTCTGCTCCTCCTGTT-3’ GAPDH-R: 5'-CCATGGTGTCTGAGCGATGT-3’

*GAPDH was used for normalization.

Amplification curves and melting curves were illustrated by CFX Maestro software (Version 1.1; Bio-Rad Laboratories). Relative mRNA levels were determined by the ΔΔCt quantification method using the CFX Maestro software. Baseline correction and threshold setting were performed using the automatic calculation offered by the software.

### Testing for residual collagenase activity in cell preparations prepared with the use of Matrase Reagent

In this experiment ADRCs were isolated from lipoaspirate from n = 3 additional subjects using the ARC system (InGeneron; a slight modification of the Transpose RT system, adapted for additional use with larger portions of adipose tissue) and Matrase Reagent following the manufacturer’s instructions for use. The resulting cell preparations were tested for collagenase activity using a commercially available assay (EnzChek Gelatinase/Collagenase Assay Kit; Invitrogen, Carlsbad, CA, USA) following the manufacturer’s instructions for use.

### Microscopy

Images were obtained using an Axio Vert.A1 microscope (Carl Zeiss Microscopy, Jena, Germany) equipped with LD A-Plan 10x/0,25 and LD A-Plan 20x/0.30 objectives (Carl Zeiss Microscopy), Leica DMC4500 camera (2560 × 1920 pixels; Leica Microsystems, Wetzlar, Germany) and Leica Application Suite software (version X 3.3.3.16958; Leica).

Final figures were constructed using Corel Photo-Paint X7 and Corel Draw X7 (both versions 17.5.0.907; Corel, Ottawa, Canada). No adjustments of contrast and brightness were made.

### Statistical analysis

Mean and standard error of the mean (SEM) were calculated for all variables. The D’Agostino and Pearson omnibus normality test was used to determine whether the distribution of the investigated variables of the Transpose RT / Matrase isolated cells and the Transpose RT / no Matrase isolated cells were consistent with a Gaussian distribution. Differences between the groups of cells were tested with nonparametric Wilcoxon matched-pairs signed rank test. In all analyses, an effect was considered statistically significant if its associated p value was smaller than 0.05. Calculations were performed with GraphPad Prism (Version 5; Graph Pad Software, San Diego, CA, USA).

## Results

### Cell yield, cell viability, number of living cells per ml lipoaspirate and cell size

**Fig 3:**
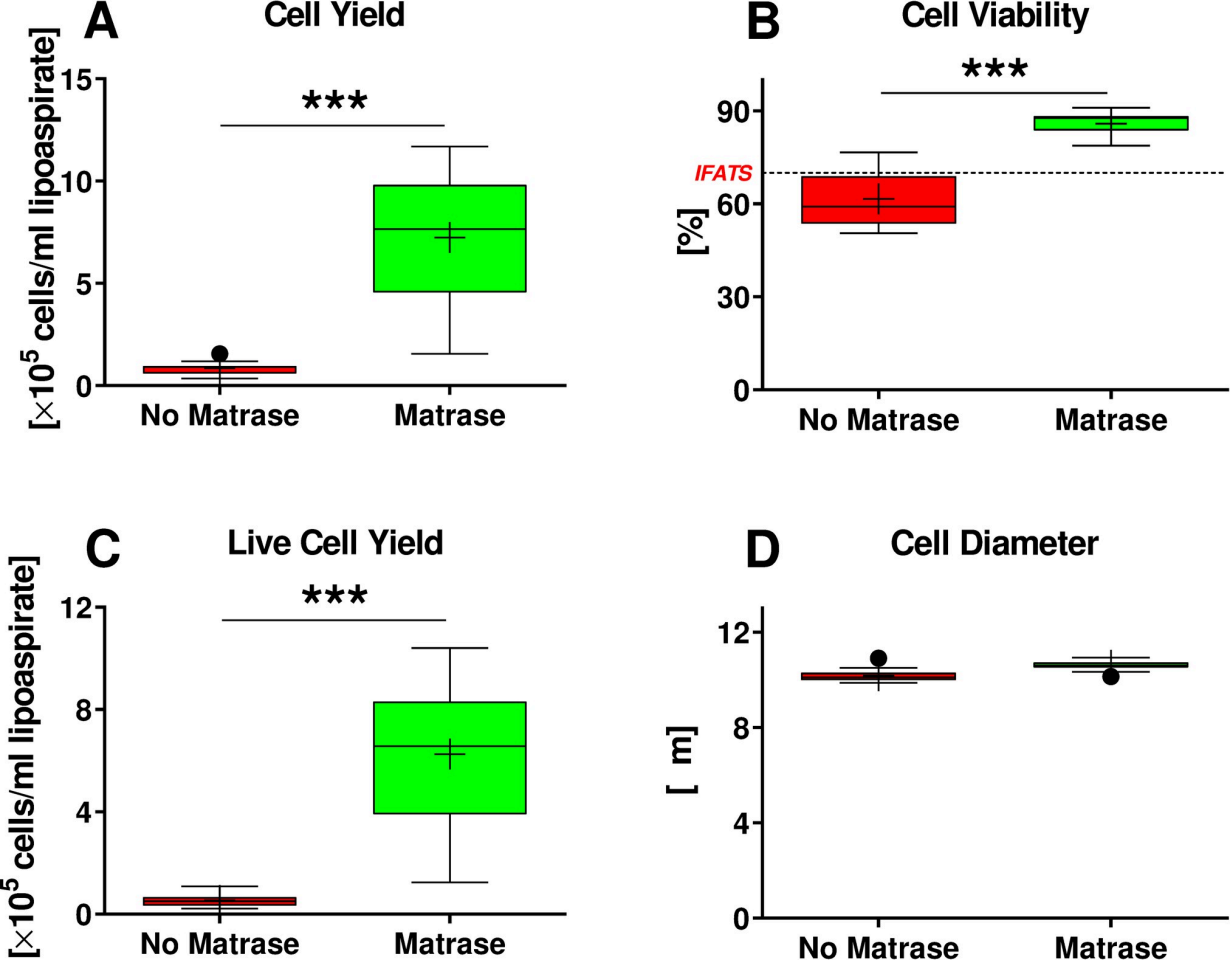
Results of quantitative analysis of Transpose RT / no Matrase isolated ADRCs and Transpose RT / Matrase isolated ADRCs. The panels show Tukey boxplots of the number of cells isolated per ml lipoaspirate (A), the relative number of viable cells (B), the number of viable cells isolated per ml lipoaspirate (C) and the diameter of cells (D) of Transpose RT / no Matrase isolated ADRCs (red bars) and Transpose RT / Matrase isolated ADRCs (green bars). In (B) the threshold of 70% viable cells established by the International Federation for Adipose Therapeutics and Science (IFATS) [73] is indicated by a dashed line. Results of Wilcoxon matched-pairs signed rank test are indicated (n = 12 paired samples each). ***, p < 0.001.

Compared to Transpose RT / no Matrase isolation, Transpose RT / Matrase isolation of ADRCs from lipoaspirate resulted in the following, statistically significant differences in the final cell suspension (all values given as mean ± SEM): (i) approximately nine times higher cell yield (7.2×10^5^ ± 0.90×10^5^ Transpose RT / Matrase isolated ADRCs per ml lipoaspirate vs. 0.84×10^5^ ± 0.10×10^5^ Transpose RT / no Matrase isolated ADRCs per ml lipoaspirate; p < 0.001; n = 12 matched pairs of samples) (Fig 3A); (ii) approximately 41% higher mean cell viability (85.9% ± 1.1% in case of Transpose RT / Matrase isolated ADRCs vs. 61.7% ± 2.6% in case of Transpose RT / no Matrase isolated ADRCs; p < 0.001; n = 12 matched pairs of samples) (Fig 3B); and (iii) approximately twelve times higher mean number of living cells per ml lipoaspirate (6.25×10^5^ ± 0.79×10^5^ Transpose RT / Matrase isolated ADRCs per ml lipoaspirate vs. 0.52×10^5^ ± 0.08×10^5^ Transpose RT / no Matrase isolated ADRCs per ml lipoaspirate; p < 0.001; n = 12 matched pairs of samples each) (Fig 3C).

Of importance, the mean relative number of viable cells obtained by Transpose RT / Matrase isolation (85.9%) exceeded the proposed minimum threshold for the viability of cells in the SVF of 70% established by the International Federation for Adipose Therapeutics and Science (IFATS) [73], whereas the mean relative number of viable cells obtained by Transpose RT / no Matrase isolation (61.7%) did not (Fig 3B).

The difference in mean cell diameter between Transpose RT / no Matrase isolated ADRCs (10.2μm ± 0.1μm) and Transpose RT / Matrase isolated ADRCs (10.6μm ± 0.1μm) was only approximately 4% and did not reach statistical significance (p = 0.05; n = 12 matched pairs of samples) (Fig 3D).

Accordingly, both the number and viability of cells in the final cell suspension were statistically significantly higher after Transpose RT / Matrase isolation of ADRCs from human adipose tissue than after Transpose RT / no Matrase isolation.

### Colony-Forming unit assay

**Fig 4:**
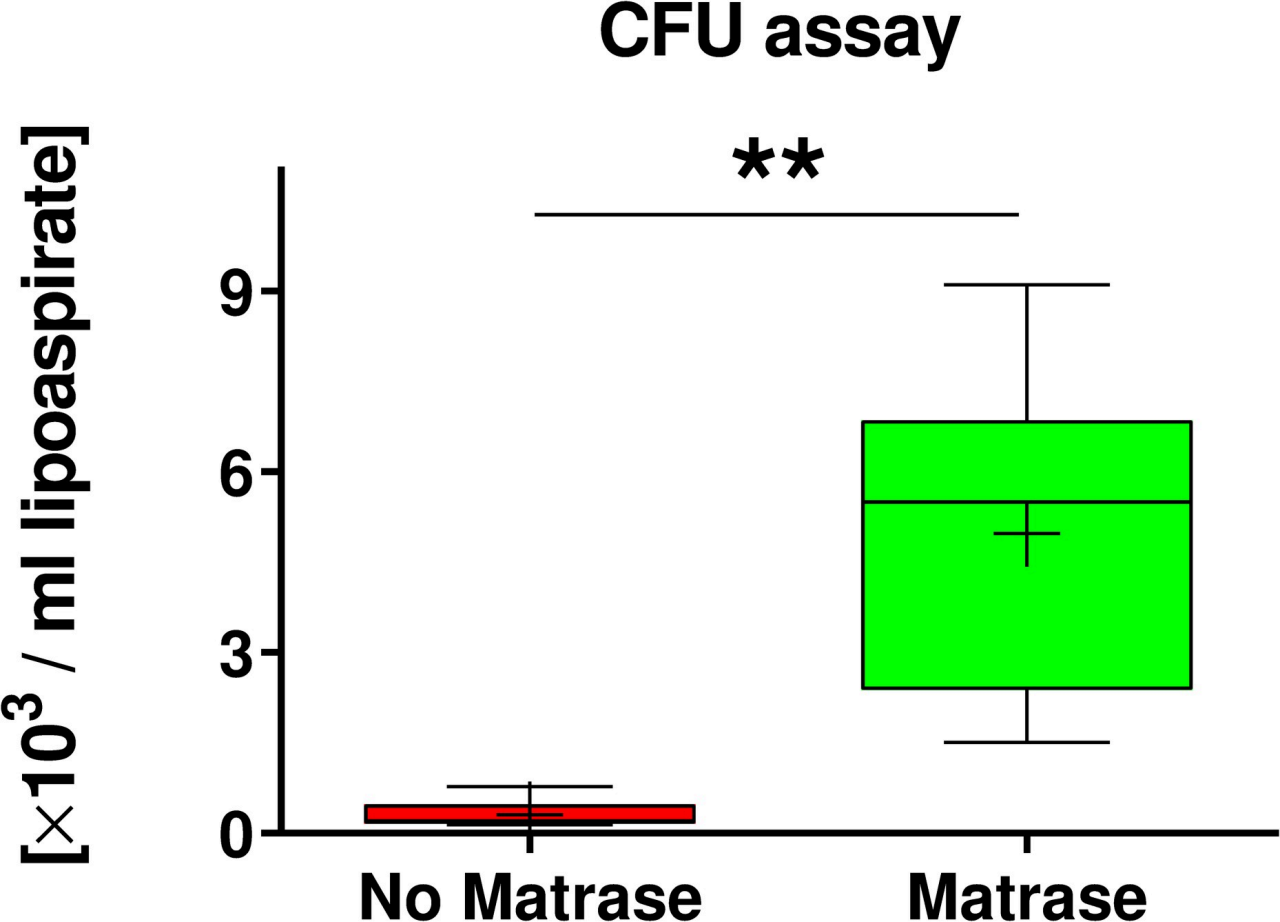
Results of colony forming unit assay. The panel shows Tukey boxplots of the number of colony forming units per ml lipoaspirate formed by ASCs derived from Transpose RT / no Matrase isolated ADRCs (red bars) and from Transpose RT / Matrase isolated ADRCs (green bars) after culturing for 14 days in complete MSC media. Results of Wilcoxon matched-pairs signed rank test are indicated (n = 10 paired samples). **, p < 0.005.

ASCs derived from Transpose RT / Matrase isolated ADRCs formed on average 16 times more CFUs per ml lipoaspirate (4973±836; mean ± SEM) than ASCs derived from Transpose RT / no Matrase isolated ADRCs (307±68) (p = 0.002; n = 10 matched pairs of samples) (Fig 4).

### Expression of regenerative cell-associated genes in adipose-derived stem cells derived from uncultured, autologous, adipose-derived regenerative cells

**Fig 5:**
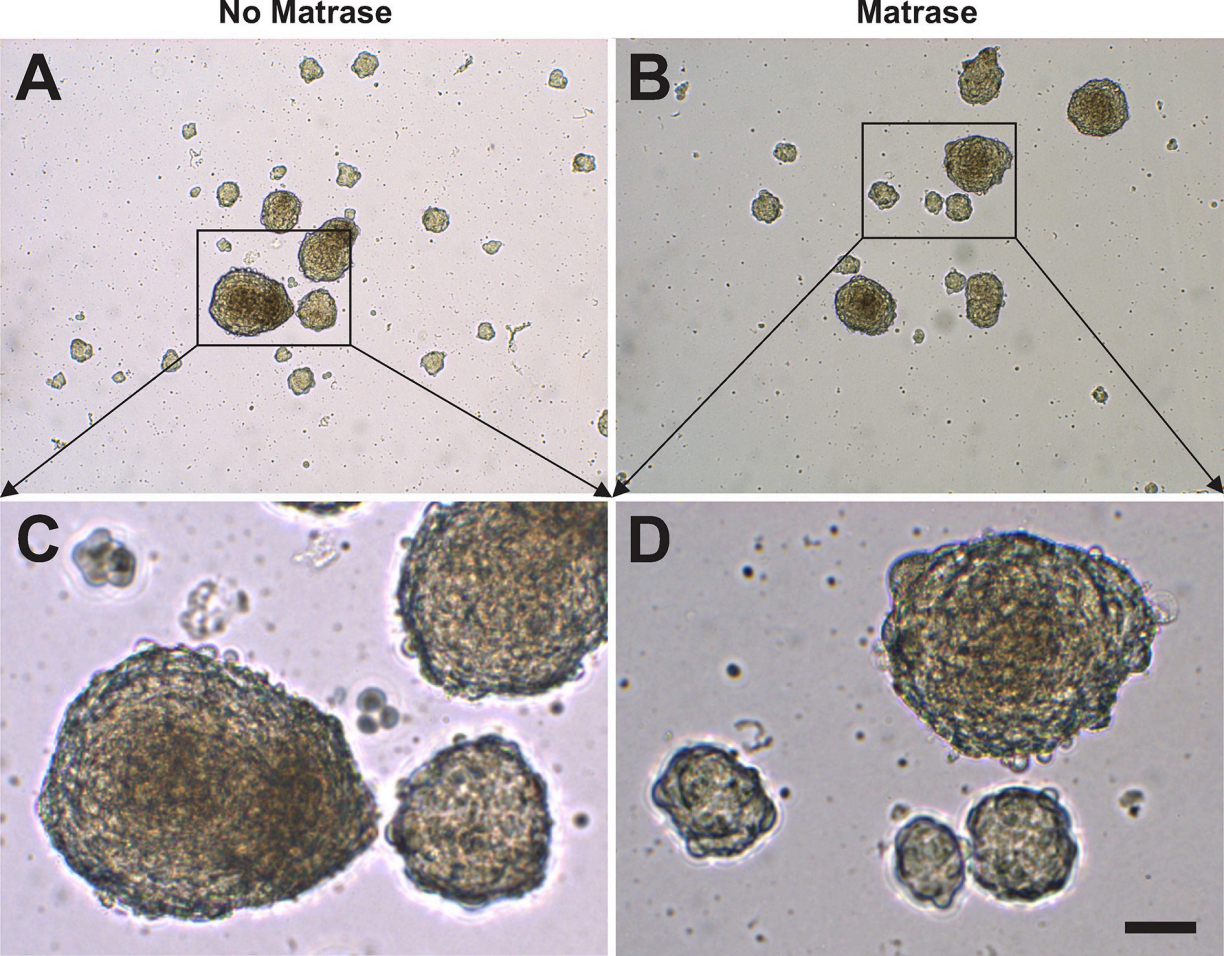
Formation of embryoid bodies. The panels show embryoid bodies that were formed after culturing ASCs derived from Transpose RT / no Matrase isolated ADRCs (A, C) and from Transpose RT / Matrase isolated ADRCs (B, D) for seven days in serum-free media. The scale bar in (D) represents 100 μm in (A, B) and 50 μm in (C, D).

**Fig 6:**
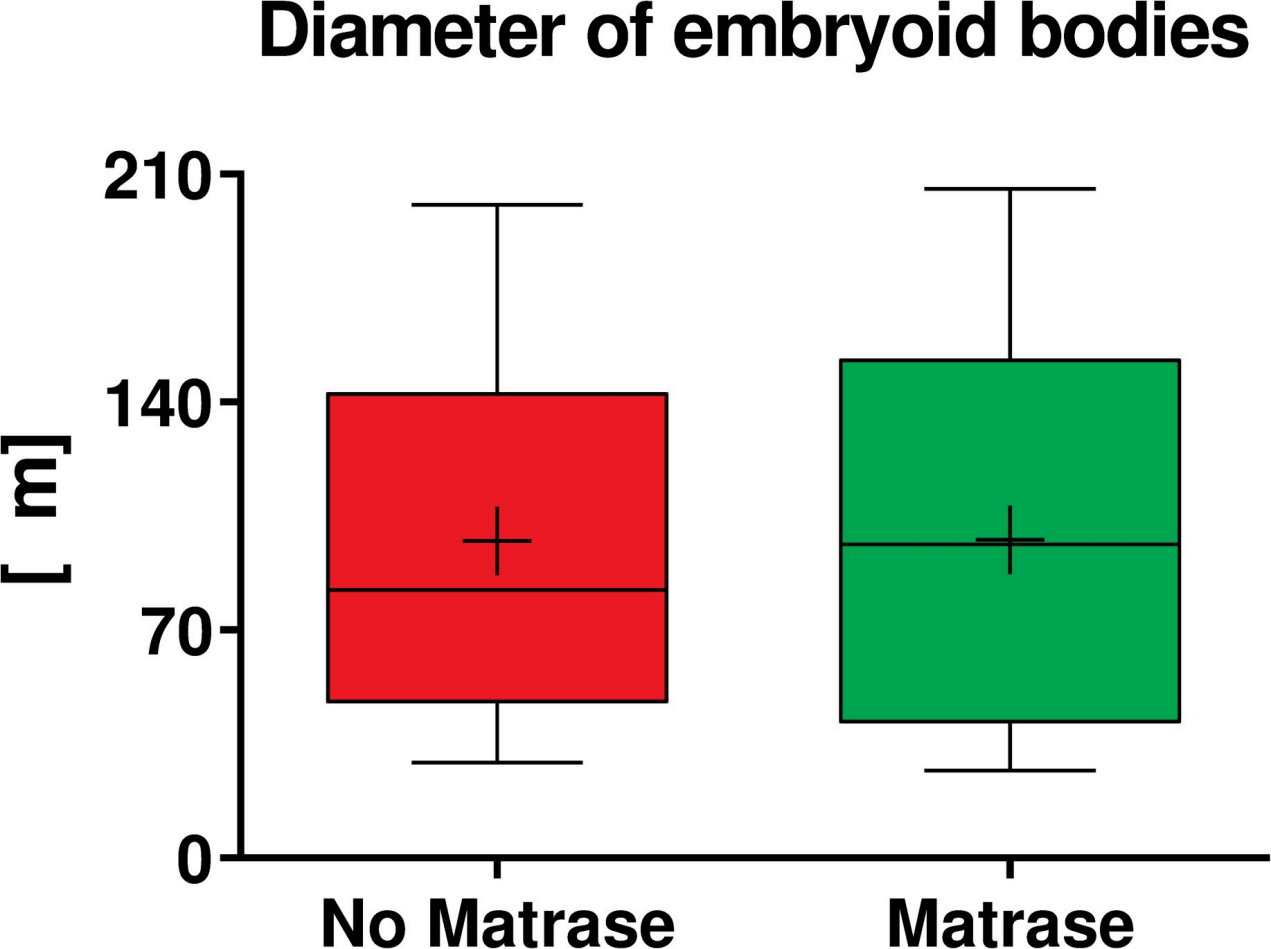
Quantitative analysis of the size of embryoid bodies. The panel shows Tukey boxplots of the diameter of embryoid bodies that were formed after culturing ASCs derived from Transpose RT / no Matrase isolated ADRCs (red bars) and from Transpose RT / Matrase isolated ADRCs (green bars) for seven days in serum-free media. The Wilcoxon matched-pairs signed rank test showed no statistically significant differences between the groups (p = 0.109; n = 4 paired samples).

Embryoid body formation was observed after culturing ASCs for seven days in serum free media (Fig 5). The embryoid bodies had a spherical appearance and defined borders. The majority of the embryoid bodies were small in diameter (<100 μm), some were medium (100–200 μm), and a few were large (>200 μm) in diameter. No statistically significant differences were observed in the formation or the size of the embryoid bodies derived from Transpose RT / no Matrase isolated ADRCs and from Transpose RT / Matrase isolated ADRCs (Fig 6).

**Fig 7:**
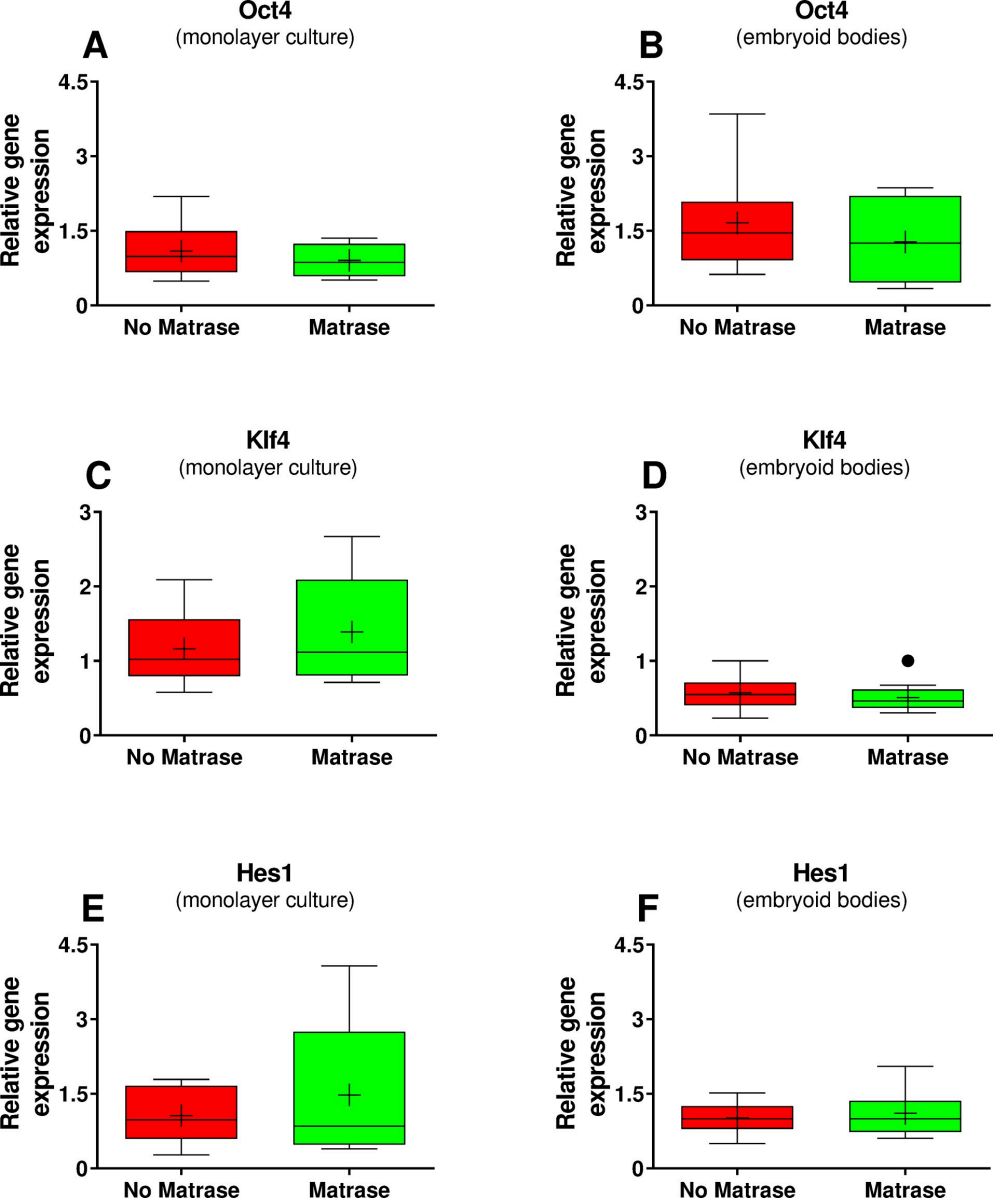
Results of gene expression analysis. The panels show Tukey boxplots of relative gene expression (arbitrary units) of Oct4 (A, B), Klf4 (C, D) and Hes3 (E, F) of ASCs in conventional monolayer cultures (A, C, E) or obtained from embryoid bodies (B, D, F) after culturing Transpose RT / no Matrase isolated ADRCs (red bars) or Transpose RT / Matrase isolated ADRCs (green bars), respectively. The Wilcoxon matched-pairs signed rank test showed no statistically significant differences between the groups (p > 0.05; n = 8 paired samples each).

Furthermore, it was found that the use of Matrase Reagent in the process of isolating cells from lipoaspirate had no statistically significant impact on the relative levels of mRNA for the regenerative cell-associated genes Oct-4, Klf4 and Hes1 for both conventional monolayer cultures (Fig 7A, 7C and 7E) and embryoid body cultures (Fig 7B, 7D and 7F) (mean and SEM of relative gene expression values as well as corresponding p-values are summarized in Table 5).

**Table 5:** Results of statistical analysis.

Variable	Transpose RT / no Matrase isolation of ADRCs	Transpose RT / Matrase isolation of ADRCs	P value
**Cell yield, cell viability, live cell yield and cell size**			
Cell yield [×10^5^]	0.84, 0.10, 12	7.24, 0.89, 12	<0.001
Cell viability [%]	61.7, 2.61, 12	85.9, 1.12, 12	<0.001
Live cell yield [×10^5^]	0.53, 0.08, 12	6.25, 0.79, 12	<0.001
Cell diameter [μm]	10.2, 0.08, 12	10.6, 0.06, 12	0.05
**Colony Forming Unit assay**			
CFUs [number]	307, 68, 10	4973, 836, 10	0.002
**Formation of embryoid bodies**			
Diameter [μm]	97.4, 11.6, 4	97.8, 11.4, 4	0.979
**Expression of regenerative cell-associated genes**			
ASCs: Oct4	1.09, 0.20, 8	0.90, 0.11, 8	0.461
ASCS: Klf4	1.16, 0.18, 8	1.39, 0.26, 8	0.195
ASCs: Hes1	1.06, 0.20, 8	1.48, 0.49, 8	0.742
EB: Oct4	1.66, 0.31, 10	1.28, 0.25, 10	0.322
EB: Klf4	0.57, 0.07, 10	0.51, 0.07, 10	0.557
EB: Hes1	1.01, 0.11, 9	1.11, 0.15, 9	0.496
**Adipogenic differentiation potential**			
OrO+ [%]	33.1, 3.17, 7	38.7, 4.72, 7	0.109

All data are provided as {mean, standard error of the mean [SEM], number of paired samples}. P-values were obtained using nonparametric Wilcoxon matched-pairs signed rank test. Cell yield and live cell yield data represent numbers of cells per ml lipoaspirate; relative gene expression data are provided in arbitrary units. Calculations were performed with GraphPad Prism (Version 5; Graph Pad Software, San Diego, CA, USA). Note: the different numbers of paired samples in the gene expression studies are related to the quantity and amount of mRNA generated for each sample. Samples that yielded either insufficient amount of mRNA or lower quality mRNA were not analyzed for the relative level of gene expression. Abbreviations: CFUs, colony forming units; ASCs, adipose-derived stem cells; EB, embryoid bodies; OrO+, No. of Oil red-O positive cells.

Accordingly, the use of Matrase Reagent in processing lipoaspirate with the InGeneron Transpose RT System did not alter expression of regenerative cell-associated genes in the final cell suspension.

### Differentiation potential of adipose-derived stem cells

#### Adipogenic differentiation potential

**Fig 8:**
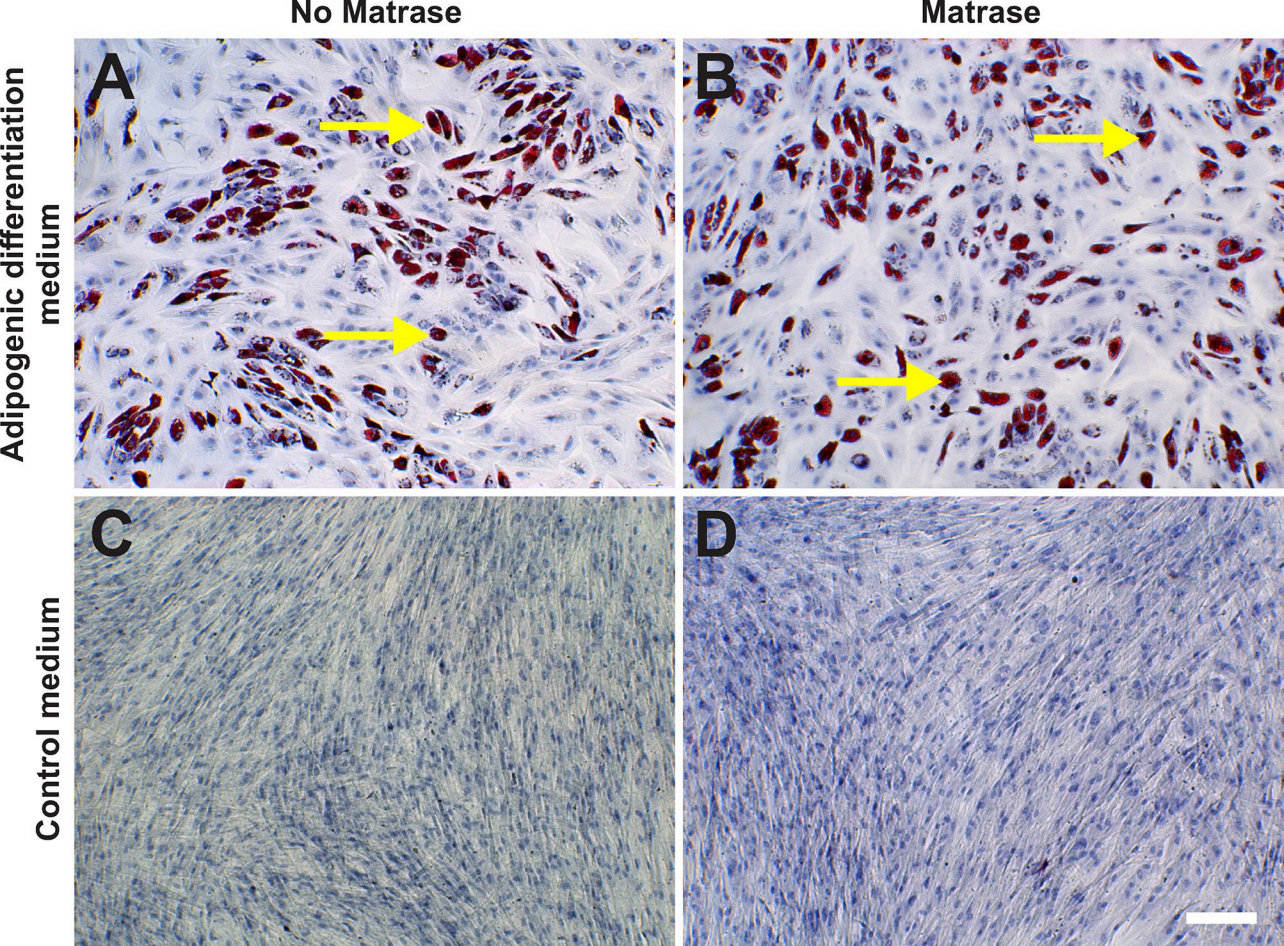
Adipogenic differentiation potential of ADRCs. The panels show the results of culturing ASCs on their 3^rd^ passage (derived from Transpose RT / no Matrase isolated ADRCs (A, C) or Transpose RT / Matrase isolated ADRCs (B, D), respectively) for two weeks in adipogenic differentiation medium (A, B) or control medium (C, D). The presence of intracytoplasmic lipids (triglycerides) was assessed with Oil red-O staining; cells were counterstained with hematoxylin. The yellow arrows indicate single Oil red-O positive cells. The scale bar in (D) represents 100 μm in (A-D).

**Fig 9:**
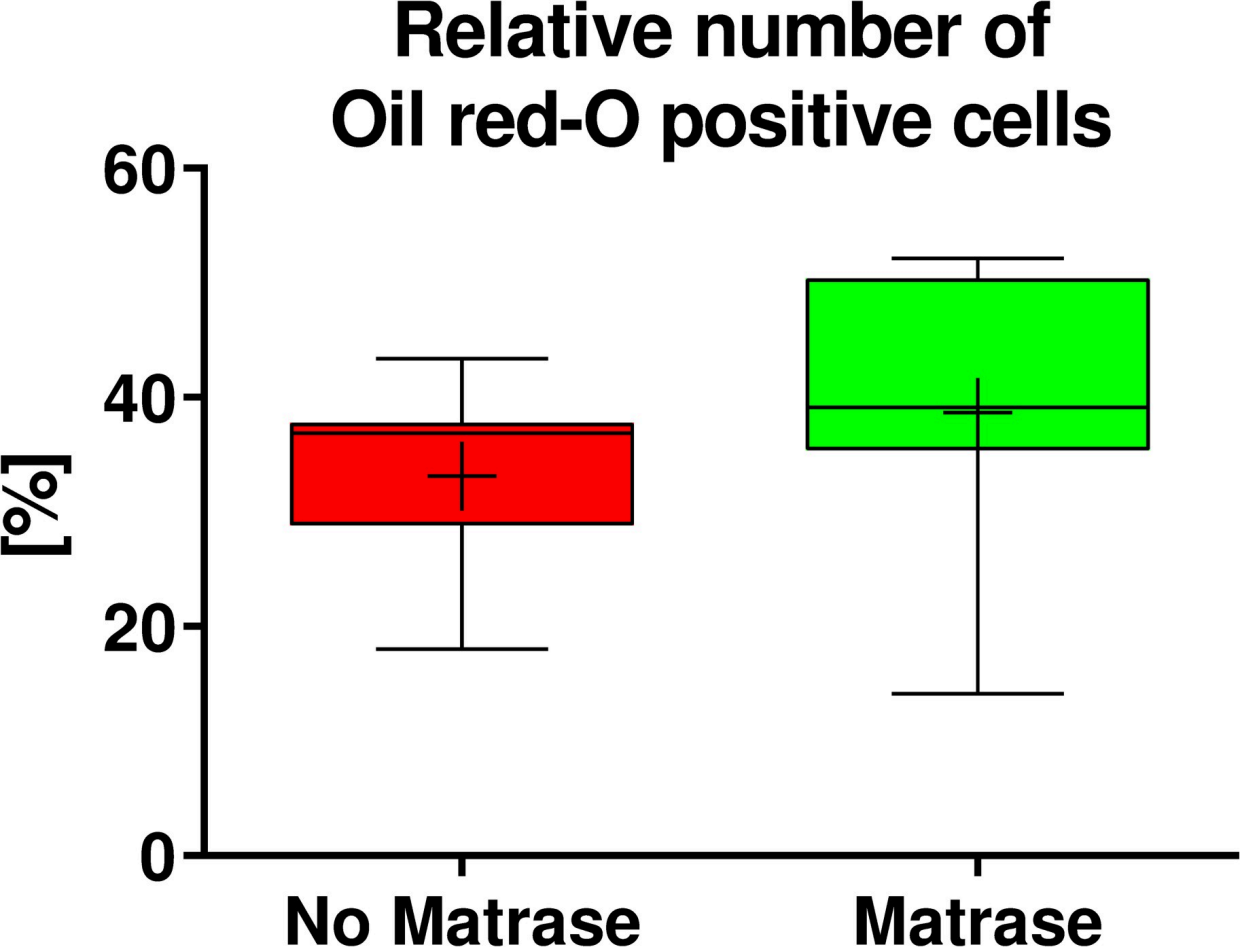
Quantitative analysis of adipogenic differentiation potential of ADRCs. The panel shows Tukey boxplots of the relative number of Oil red-O positive cells obtained after culturing ASCs on their 3rd passage (derived from Transpose RT / no Matrase isolated ADRCs (red bars) or Transpose RT / Matrase isolated ADRCs (green bars), respectively) for two weeks in adipogenic differentiation medium. The Wilcoxon matched-pairs signed rank test showed no statistically significant differences between the groups (p = 0.109; n = 7 paired samples).

ASCs on their 3^rd^ passage (derived from both Transpose RT / Matrase isolated ADRCs and Transpose RT / no Matrase isolated ADRCs) were cultured for two weeks in adipogenic differentiation medium or control medium. Then, the presence of intracytoplasmic lipids (triglycerides) was assessed with Oil red-O staining, and relative numbers of Oil red-O positive cells were evaluated. It was found that the use of Matrase Reagent in the process of isolating ADRCs from lipoaspirate had no impact on the visual appearance of the cells after induction of adipogenic differentiation (Fig 8), and no statistically significant impact on the relative number of Oil red-O positive cells (Fig 9).

#### Osteogenic differentiation potential

**Fig 10:**
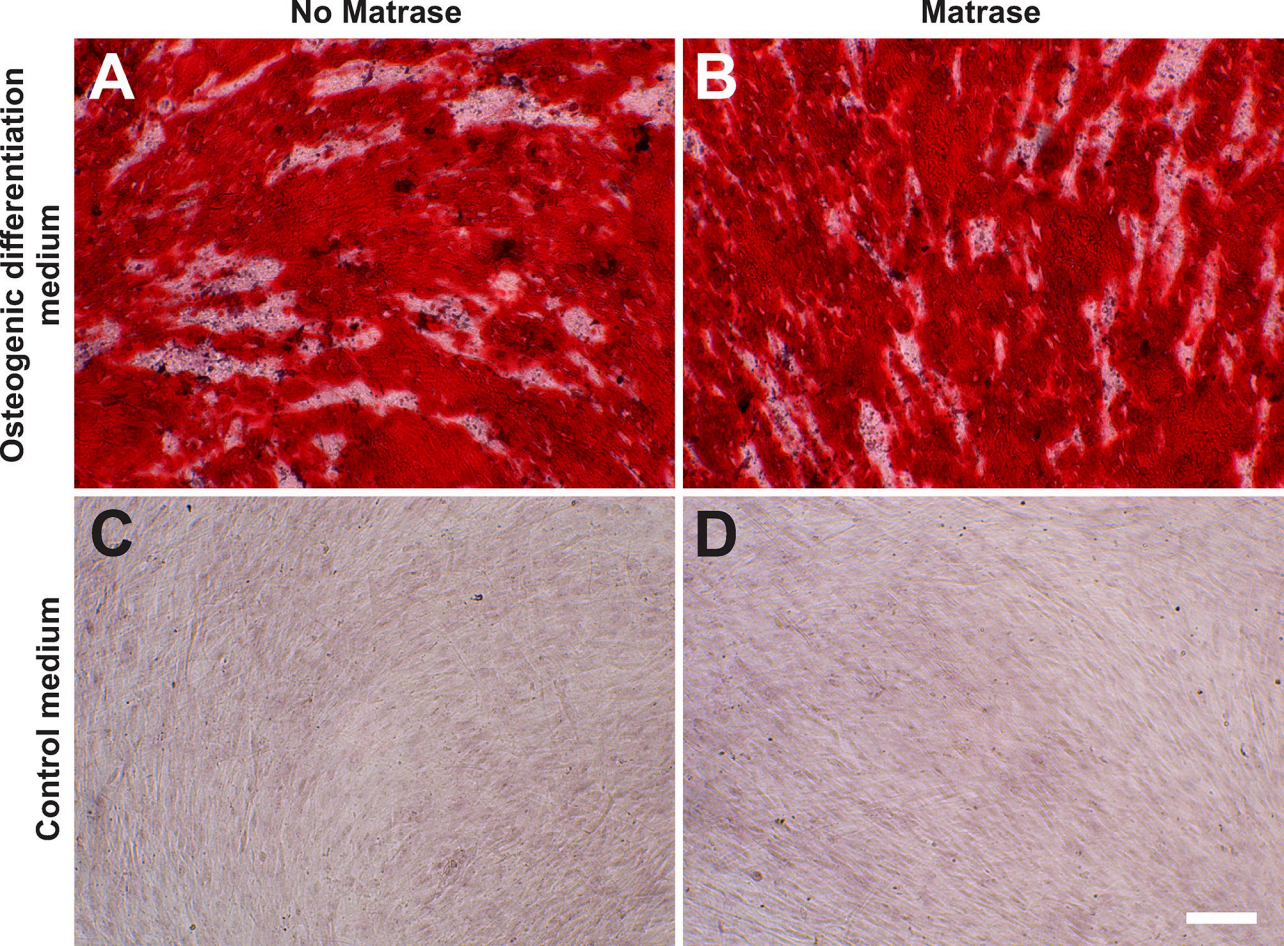
Osteogenic differentiation potential of ADRCs. The panels show the results of culturing ASCs on their 3^rd^ passage (derived from Transpose RT / no Matrase isolated ADRCs (A, C) or Transpose RT / Matrase isolated ADRCs (B, D), respectively) for two weeks in osteogenic differentiation medium (A, B) or control medium (C, D). The presence of calcific deposits was investigated with Alizarin red staining; cells were counterstained with hematoxylin. Cells of an osteogenic lineage are stained bright to deep red and easily visible as dense red patches. The scale bar in (D) represents 100 μm in (A-D).

ASCs on their 3^rd^ passage (derived from both Transpose RT / Matrase isolated ADRCs and Transpose RT / no Matrase isolated ADRCs) were cultured for two weeks in osteogenic differentiation medium or control medium. Then, the presence of calcific deposits was investigated with Alizarin red staining. It was found that the use of Matrase Reagent in the process of isolating ADRCs from lipoaspirate had no impact on the visual appearance of the cells after induction of osteogenic differentiation (Fig 10).

#### Hepatogenic differentiation potential

**Fig 11:**
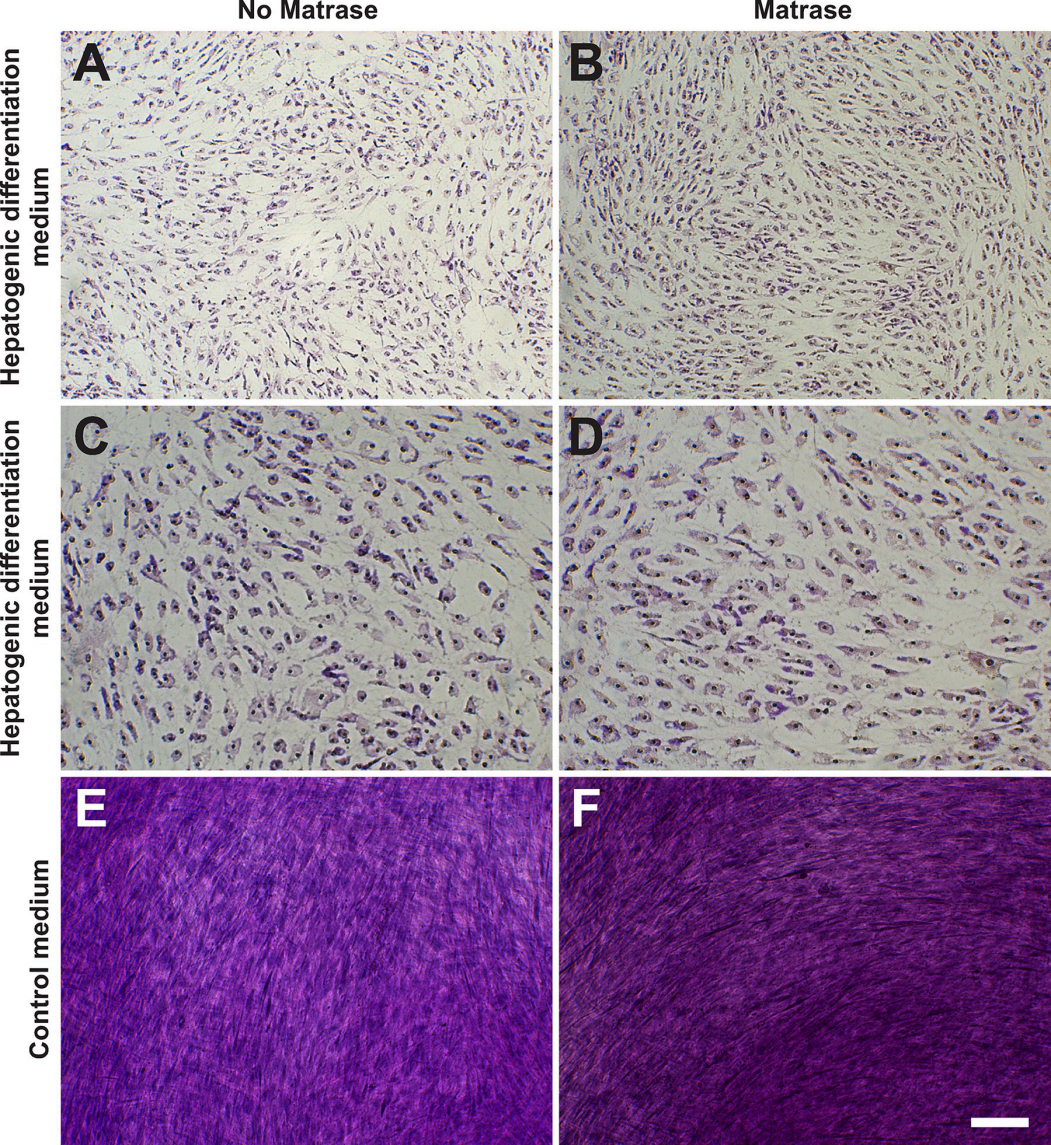
Hepatogenic differentiation potential of ADRCs. The panels show the results of culturing ASCs on their 3^rd^ passage (derived from Transpose RT / no Matrase isolated ADRCs (A, C, E) or Transpose RT / Matrase isolated ADRCs (B, D, F), respectively) for ten days in hepatogenic differentiation medium (A-D) or control medium (E, F). The presence of structures containing a high proportion of carbohydrate macromolecules (glycogen, glycoprotein and proteoglycans) was investigated with Periodic Acid Schiff staining; cells were counterstained with hematoxylin. As a result of induction of hepatogenesis the morphology of the cells changed from a fibroblastic spindle shape to a rather polygonal shape typically associated with hepatocytes. The scale bar in (F) represents 100 μm in (A, B, E, F) and 50 μm in (C, D).

ASCs on their 3^rd^ passage (derived from both Transpose RT / Matrase isolated ADRCs and Transpose RT / no Matrase isolated ADRCs) were cultured for ten days in hepatogenic differentiation medium or control medium. Then, the presence of structures containing a high proportion of carbohydrate macromolecules (glycogen, glycoprotein and proteoglycans) was investigated with Periodic Acid Schiff staining. It was found that the use of Matrase Reagent in the process of isolating ADRCs from lipoaspirate had no impact on the visual appearance of the cells after induction of hepatogenic differentiation (Fig 11).

#### Neurogenic differentiation potential

**Fig 12:**
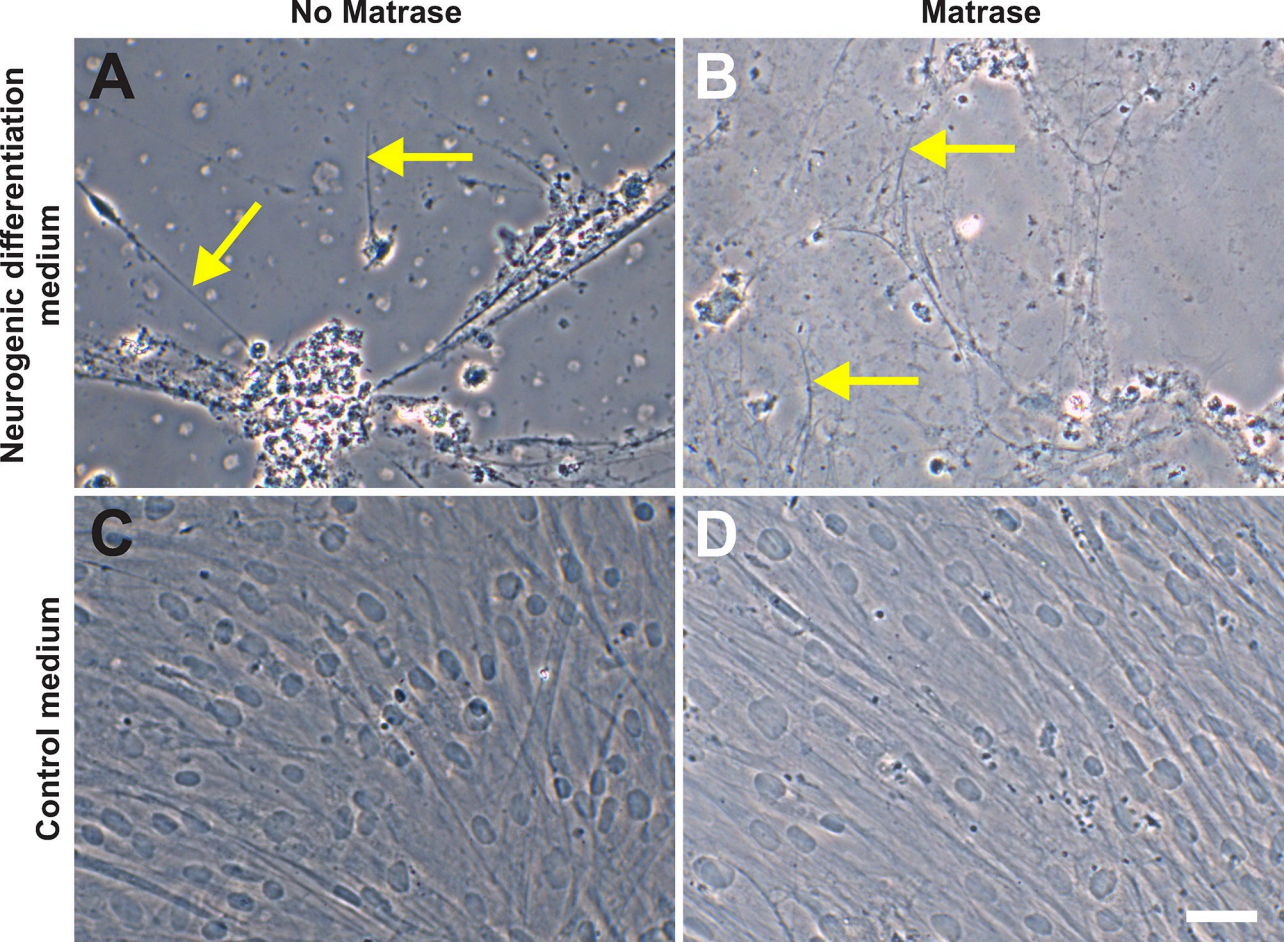
Neurogenic differentiation potential of ADRCs (1). The panels show the results of culturing ASCs on their 6^th^ passage (derived from Transpose RT / no Matrase isolated ADRCs (A, C) or Transpose RT / Matrase isolated ADRCs (B, D), respectively) for three weeks in neurogenic differentiation medium (A, B) or control medium (C, D). Cells were imaged with phase contrast microscopy. As a result of induction of neurogenesis, the cells developed characteristic, slender processes (arrows in A, B). The scale bar in (D) represents 50 μm in (A-D).

**Fig 13:**
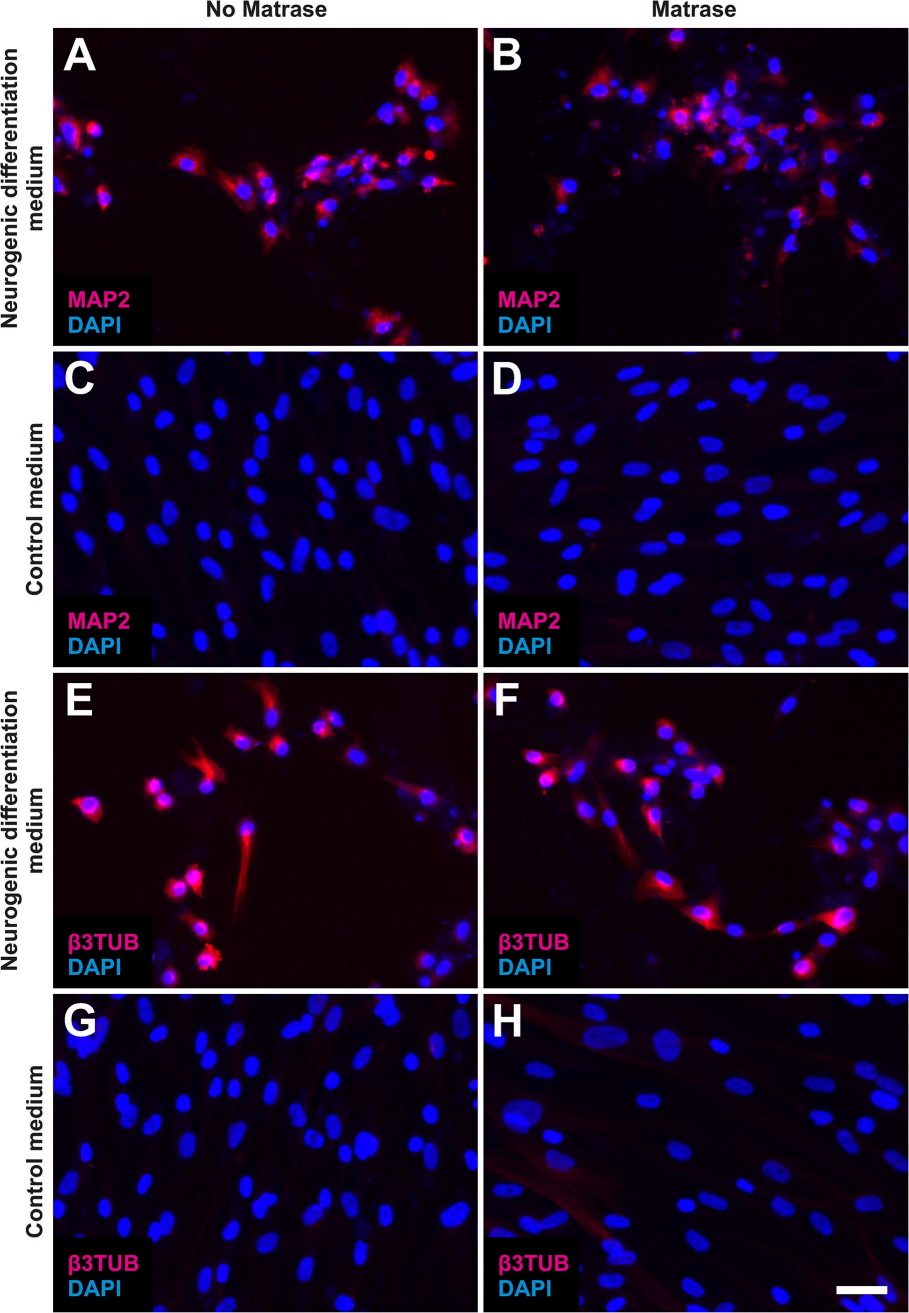
Neurogenic differentiation potential of ADRCs (2). The panels show the results of culturing ASCs on their 6^th^ passage (derived from Transpose RT / no Matrase isolated ADRCs (A, C, E, G) or Transpose RT / Matrase isolated ADRCs (B, D, F, H), respectively) for three weeks in neurogenic differentiation medium (A, B, E, F) or control medium (C, D, G, H). Cells were processed with immunofluorescence for the detection of respectively microtubule-associated protein 2 (MAP2) (A-D) or beta III Tubulin (β3TUB) (E-H), and were counterstained with DAPI. As a result of induction of neurogenesis, the cells expressed both MAP2 (A, B) and β3TUB (E, F). The scale bar in (H) represents 50 μm in (A-H).

**Fig 14:**
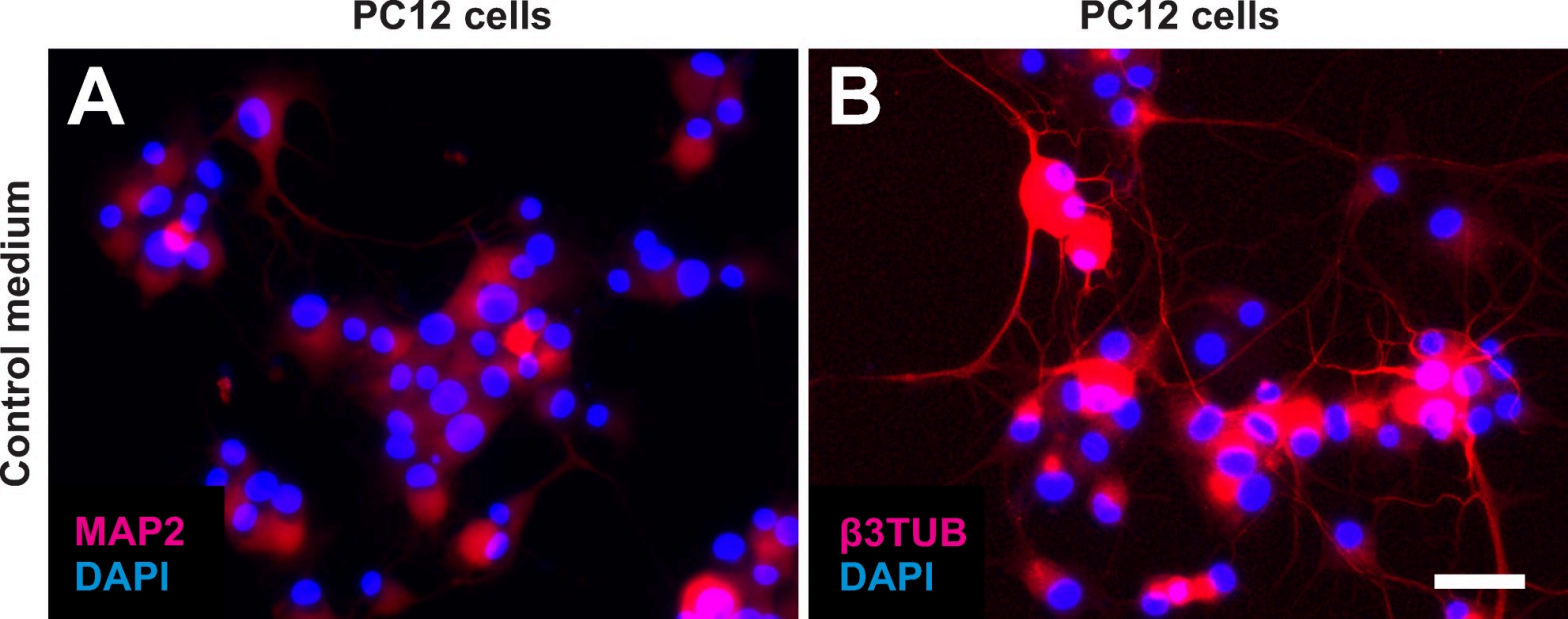
Expression of microtubule-associated protein 2 (MAP2) and beta III tubulin (β3TUB) by PC12 cells as positive control. The panels show the results of culturing PC12 cells for 6 days in neurogenic culture medium. Cells were processed with immunofluorescence for the detection of respectively MAP2 (A) or β3TUB (B), and were counterstained with DAPI. The scale bar in (B) represents 50 μm in (A, B).

ASCs on their 6^th^ passage (derived from both Transpose RT / Matrase isolated ADRCs and Transpose RT / no Matrase isolated ADRCs) were cultured for three week in neurogenic differentiation medium or control medium. Then, the morphology of the cells was investigated with phase contrast microscopy, and expression of MAP2 and β3TUB with immunofluorescence. It was found that as a result of induction of neurogenesis, the cells showed key characteristics of a neuronal phenotype, i.e., slender processes (Fig 12) and expression of both MAP2 and β3TUB (Fig 13). PC12 cells served as positive controls (Fig 14). The use of Matrase Reagent in the process of isolating ADRCs from lipoaspirate had no impact on the visual appearance of the cells after induction of neurogenic differentiation (Figs 12 and 13).

### Residual collagenase activity in cell preparations prepared with the use of Matrase Reagent

**Fig 15:**
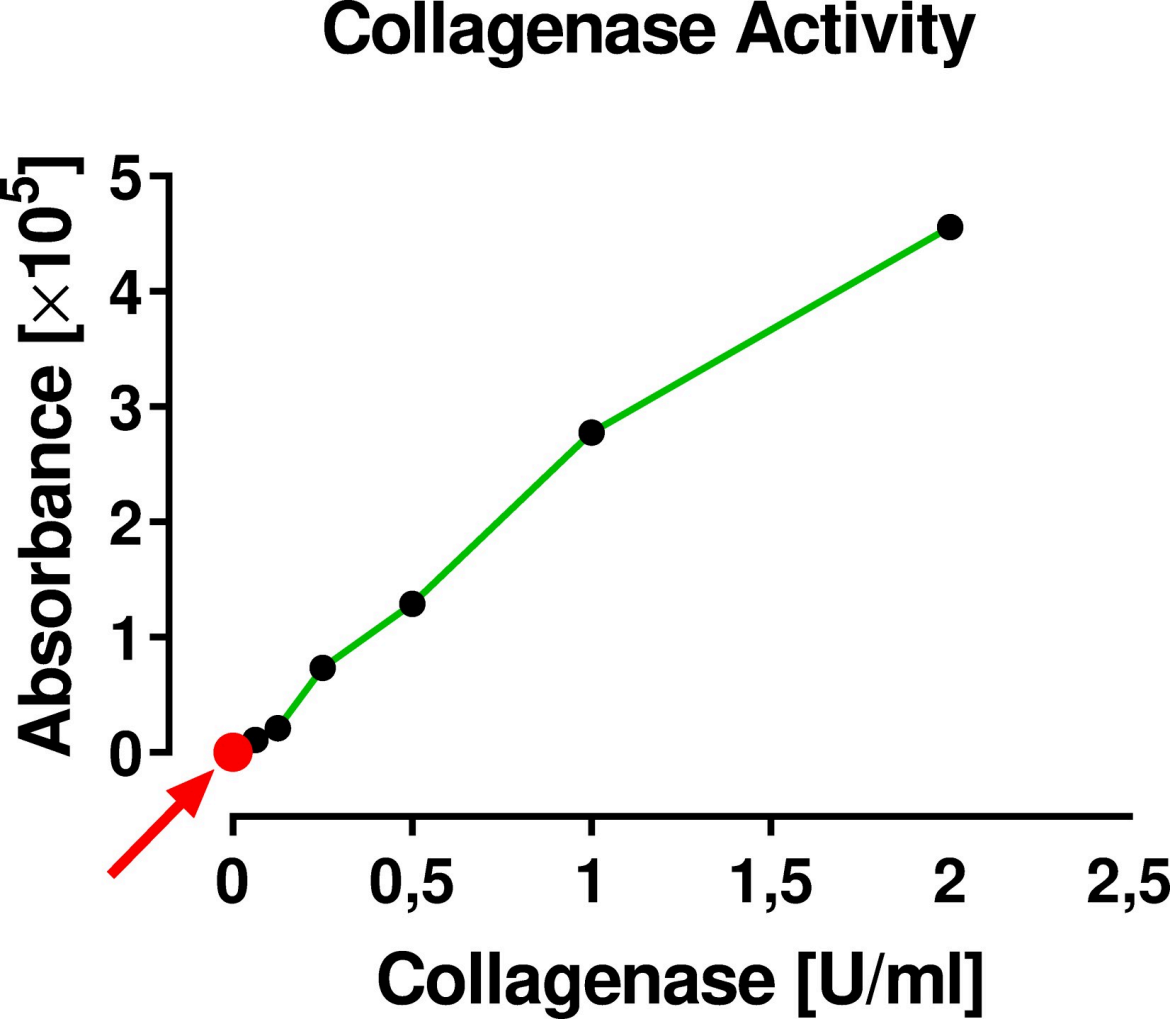
Measurement of collagenase activity in cell preparations that were prepared with the use of Matrase Reagent following the manufacturer’s instructions for use. No collagenase activity was detected in cell preparations (the red dot at Y = X = 0 [indicated by the red arrow] represents the mean of two independent measurements for each sample). The standard curve for the used assay (EnzChek Gelatinase/Collagenase Assay Kit E12055; Invitrogen) is depicted by the green line. Because this high-sensitivity assay can detect enzyme activity down to a minimum concentration of 2×10^−3^ U/ml, activity of the enzyme in the final cell preparation was not present or less than two-thousands of a unit per ml.

It was found that the collagenase activity was below the detection limit of the used assay (Fig 15).

## Discussion

Aronowitz and colleagues [46] proposed to judge a system or method for isolating ADRCs from adipose tissue by the following factors: nucleated cell count, nucleated cells per milliliter of tissue processed, cellular viability, level of residual enzymatic activity, data from flow cytometry and CFU-F assay, infection control, ease of use, cost to operate and processing time.

There are currently several systems offered and methods described in the literature for clinical therapeutic usage (or that are under clinical evaluation) that process adipose tissue with (Table 1) or without (Table 2) the use of enzymes. It was the aim of this study to evaluate the effect on cells, efficiency and safety regarding cell viability in direct comparison between processing human lipoaspirate with or without the use of an enzyme under otherwise identical processing conditions. InGeneron’s Transpose RT system utilizes a proprietary method for isolating ADRCs from adipose tissue. Specifically, this method uses the enzymatic activity of Matrase Reagent to release the cells from the extracellular matrix.

We observed a substantial, statistically significantly lower cell yield and cell viability in the final cell suspension isolated by just mechanical processing of human lipoaspirate without the use of Matrase Reagent, due to less efficient release of cells from the extracellular matrix when no enzyme was used. Specifically, the live cell yield of Transpose RT / Matrase isolated ADRCs was approximately twelve times higher in the final cell suspension than the live cell yield of Transpose RT / no Matrase isolated ADRCs. Of importance, the mean relative number of 86% viable Transpose RT / Matrase isolated ADRCs exceeded the proposed minimum threshold for viability of cells in the SVF of 70% established by IFATS [73], whereas the mean relative number of 61% viable Transpose RT / no Matrase isolated ADRCs did not.

**Fig 16:**
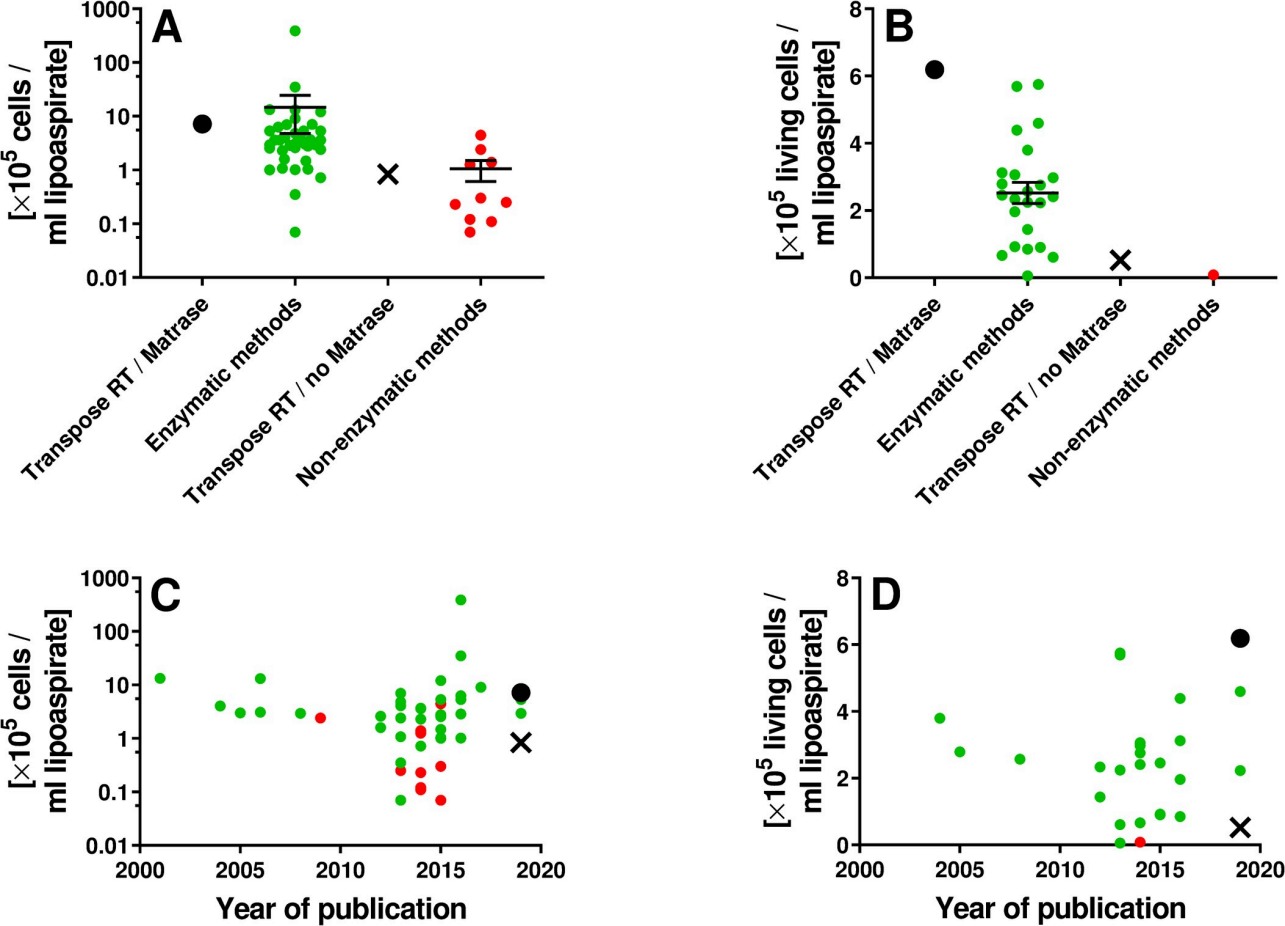
Cell yield and live cell yield obtained with Transpose RT / Matrase isolation of ADRCs and Transpose RT / no Matrase isolation of ADRCs compared with corresponding data reported in the literature. Panels A and B show individual data as well as mean ± standard error of the mean of cell yield (A) and live cell yield (B) obtained with Transpose RT / Matrase isolation of ADRCs (black dots), enzymatic methods for isolating ADRCs reported in the literature (listed in Table 1) (green dots), Transpose RT / no Matrase isolation of ADRCs (black crosses) and non-enzymatic methods for isolating ADRCs reported in the literature (listed in Table 2) (red dots). Panels C and D show the same data as a function of the year of publication.

In Fig 16 cell yield and live cell yield data obtained in this study for Transpose RT / Matrase isolated ADRCs and Transpose RT / no Matrase isolated ADRCs are compared with corresponding data reported in the literature (c.f. Tables 1 and 2). For only six out of 39 enzymatic methods (15%) a higher cell yield was reported than for Transpose RT / Matrase isolated ADRCs found in this study (Fig 16A). However, the cell yield reported for one of these enzymatic methods (387×10^5^) [63] was approximately 80 times higher than the average cell yield reported for all other 38 methods listed in Table 1 (4.8×10^5^). Because the authors of [40] did not compare their extraordinarily high cell yield data with any of the data listed in Table 1, the cell yield reported in [40] should be treated with caution. Moreover, no cell viability data were provided in those six studies [40, 42, 60–63] that reported higher cell yield for enzymatic methods than found in this study for Transpose RT / Matrase isolated ADRCs. Accordingly, live cell yield could only be calculated for 25 out of the 39 enzymatic methods (64%) listed in Table 1, and live cell yield found in this study for Transpose RT / Matrase isolated ADRCs was higher than live cell yield calculated for any of these 25 enzymatic methods (Fig 16B). Likewise, live cell yield could only be calculated for one out of the ten enzymatic methods (10%) listed in Table 2, and live cell yield found in this study for Transpose RT / no Matrase isolated ADRCs was higher (Fig 16B).

For considerations of clinical usage, these findings have an important impact: the significantly lower viability of cells isolated just mechanically without Matrase Reagent means that nearly 40% of the cells that would be transferred to a patient are not viable. In this regard is of note that Aronowitz and colleagues [46] already pointed out that the viability of the cells in the final cell suspension is a very important clinical factor because nonviable cells may provide no therapeutic value but could potentially lead to excess localized inflammation at the treatment site because of excessive cellular debris [46]. This should also be taken into account when considering the use of a non-enzymatic method for isolating ADRCs from human adipose tissue. As pointed out above, for nine out of the ten non-enzymatic methods for isolating ADRCs listed in Table 2 no cell viability data were provided, and both the non-enzymatic method described in [43] and Transpose RT / no Matrase isolation of ADRCs described in this study resulted in cell viability data that were below the threshold of 70% viable cells established by IFATS [73].

Cells isolated in this study from lipoaspirate were able to form CFUs independent of enzyme usage, indicating the presence of stem cells in both methods of isolating ADRCs from adipose tissue. However, Transpose RT / Matrase isolated ADRCs formed on average 16 times more CFUs per ml lipoaspirate than Transpose RT / no Matrase isolated ADRCs. These results are in line with previously reported studies comparing enzymatic to non-enzymatic extraction of ADRCs from lipoaspirate [58], as well as with results of cells isolated from adipose tissue or just from the tumescence fluid portion [61]. These data implicate that in order to even match the number of spheroids formed from Transpose RT / Matrase isolated ADRCs, 16 times more adipose tissue would be required as starting material for processing without enzyme. In other words, instead of 100 g patient derived adipose tissue for enzymatic processing, 1600 g for the just mechanical process would be required. This could mean a more complex recovery procedure and potentially higher morbidity, especially in patients at increased risk of bleeding. In this regard it was speculated in a recent study addressing treatment of osteoarthritis [74] that augmenting non-enzymatically isolated ADRCs with platelet rich plasma (PRP) could overcome the increased need for adipose tissue. However, no cell yield and no cell viability of the non-enzymatically isolated ADRCs were reported in [74]; no comparison of application of ADRCs with or without simultaneous application of PRP was performed; and clinical outcome was not validated with magentic resonance imaging, arthroscopy and microscopic evaluation of biopsies in [74].

Aside from substantial differences in cell yield, cell viability and live cell yield, Transpose RT / Matrase isolated ADRCs showed no statistically significant differences in the expression of regenerative cell-associated genes Oct4, Hes1 and Klf4 compared to Transpose RT / no Matrase isolated ADRCs. Besides this, both Transpose RT / Matrase isolated ADRCs and Transpose / no Matrase isolated ADRCs were able to differentiate into all three germ layers (i.e., into the adipogenic, osteogenic, hepatogenic and neurogenic lineages). This was in line with all enzymatic and non-enzymatic methods for isolating ADRCs listed in Tables 1 and 2 for which differentiation into all three germ layers was investigated.

In a joined position statement published by IFATS and the International Society for Cellular Therapy (ISCT) in 2013 regarding SVF and culture-expanded ASCs it was stated that primary stable positive surface markers for stromal cells are CD13, CD29, CD34 (>20%), CD44, CD73 and CD90 (>40%), whereas primary negative surface markers for stromal cells are CD31 (<20%) and CD45 (<50%) [73]. Furthermore, at least 20% of the SVF would contain a stromal cell population that is immunopositive for the surface marker CD34 and immunonegative for the surface markers CD31, CD45 and CD235a (i.e., CD31^-^/CD34^+^/CD45^-^/CD235a^-^ cells) [73]. This statement was based on an earlier position statement published by ISCT in 2006 that described the following minimal criteria for defining multipotent mesenchymal stromal cells (MSCs): being adherent to plastic, expressing the surface markers CD73, CD90 and CD105, and having the ability to differentiate into osteoblasts, adipocytes and chondrocytes [75]. It should be pointed out that a major shortcoming of this definition of multipotent MSCs is the fact that, for example, fibroblasts are also adherent to plastic and express the surface markers CD73, CD90 and CD105, without having the ability to transdifferentiate into other lineages or being MSCs [71]. Furthermore, the true pluripotent stem cells do not yet express CD73, CD90 and CD105 [76]. Rather, expression of cell surface markers is a dynamic process. For example, when cultured in fetal bovine serum or platelet lysate culture media, MSCs can turn on new surface markers [76]. Alternatively, MSCs in culture can lose their surface marker expression, such as for example the loss of the previously expressed progenitor marker CD34 or the endothelial progenitor marker CD31 [76].

Nevertheless, Tables 6 and 7 summarize the relative amount of ADRCs expressing the surface markers CD13, CD29, CD34, CD44, CD73, CD90, CD31 and CD45 as reported in all studies describing enzymatic and non-enzymatic methods for isolating ADRCs listed in Tables 1 and 2 (note that in some studies surface markers were investigated but relative amounts of ADRCs expressing a certain surface marker or a combination of surface markers were not provided). The data summarized in Tables 6 and 7 demonstrate that (i) for only very few methods [49, 53, 54, 58] the relative amount of CD34^+^ ADRCs was determined, with substantial variation among methods (range, 35% - 81%); (ii) for most methods CD34 was determined together with at least one other surface marker, resulting in a range of published data between 0.8% (CD34^+^/CD90^-^/CD31^-^/CD45^-^/CD105^-^/CD146^+^ cells; [51]) and 44% (CD34^+^/CD31^-^/CD45^-^ cells; [41]); (iii) the relative amount of CD45^+^ ADRCs varied between 6% [42] and 50% [54] for enzymatic methods, and between 8% [42] and 82% [58] for non-enzymatic methods; (iv) for only few methods the relative amounts of CD13^+^ cells, CD29^+^ cells, CD44^+^ cells, CD73^+^ cells, CD90^+^ cells and CD31^+^ cells were determined; and (v) for no any method the relative amount of CD31^-^/CD34^+^/CD45^-^/CD235a^-^ cells (as proposed in [73]) was determined.

**Table 6:** Relative amount of ADRCs expressing certain surface markers as reported in studies describing non-enzymatic methods for isolating ADRCs.

R	Y	CY	CD13	CD29	CD34	CD44	CD73	CD90	CD31	CD45
[39]	2015	**0.0**	--	--	--	--	--	--	--	--
[41]	2013	**0.07**	--	--	28^(a)^	--	--	--	^(a)^	^(a)^
[41]	2013	**0.35**	--	--	26^(a)^	--	--	--	^(a)^	^(a)^
[44]	2014	**0.72**	--	--	--	--	--	--	--	--
[45]	2015	**1.00**	--	--	20^(b)^	--	--	--	4^(c)^	--
[46]	2016	**1.01**	--	--	11^(a)^	--	--	--	^(a)^	^(a)^
[45]	2015	**1.03**	--	--	25^(b)^	--	--	--	5^(c)^	--
[41]	2013	**1.07**	--	--	22^(a)^	--	--	--	^(a)^	^(a)^
[45]	2015	**1.47**	--	--	--	--	--	--	--	--
[47]	2012	**1.60**	--	--	42^(b)^	--	--	--	--	36
[48]	2014	**2.30**	--	--	--	--	60^(d)^	^(d)^	--	^(d)^
[41]	2013	**2.41**	--	--	44^(a)^	--	--	--	^(a)^	^(a)^
[39]	2015	**2.55**	--	--	--	--	--	--	--	--
[47]	2012	**2.60**	--	--	41^(b)^	--	--	--	--	34
[46]	2016	**2.85**	--	--	9^(a)^	--	--	--	^(a)^	^(a)^
[49]	2019	**2.93**	33	76	60	60	48	68	45	23
[50]	2015	**2.79**	--	--	36^(e)^	--	--	^(e)^	--	5^(f)^
[51]	2008	**2.95**	--	--	0.8^(g)^	--	--	--	8^(h)^	--
[52]	2005	**3.00**	--	--	--	--	--	--	--	--
[53]	2006	**3.08**	37	47	60	64	25	55	22	--
[54]	2014	**3.60**	--	--	35	--	--	--	8^(i)^	50
[55]	2014	**3.60**	--	--	--	--	--	--	--	--
[43]	2014	**3.60**	--	--	--	--	--	--	--	--
[56]	2014	**3.68**	--	--	--	--	--	--	--	--
[23]	2004	**4.04**	--	--	--	--	--	--	--	--
[57]	2013	**4.07**	--	--	--	--	--	--	--	--
[58]	2013	**4.80**	--	90	81	6	37	81	--	28
[42]	2015	**5.34**	--	--	20^(a)^	--	--	--	--	6
[49]	2019	**5.35**	48	89	67	68	59	76	55	31
[46]	2016	**5.36**	--	--	9^(a)^	--	--	--	^(a)^	^(a)^
[46]	2016	**6.25**	--	--	7^(a)^	--	--	--	^(a)^	^(a)^
[59]	2013	**7.01**	--	--	18^(b)^	--	--	--	27^(i)^	--
[59]	2013	**7.02**	--	--	43^(b)^	--	--	--	25^(i)^	--
[60]	2017	**9.06**	--	--	--	--	--	--	--	--
[42]	2015	**12.1**	--	--	19^(a)^	--	--	--	--	13
[61]	2006	**13.1**	--	--	28^(j)^	--	--	--	4^(k)^	30^(l)^
[62]	2001	**13.3**	--	--	--	--	--	--	--	--
[63]	2016	**35.0**	--	--	--	--	--	--	--	--
[40]	2016	**387**	--	--	--	--	--	22^(m)^	^(m)^	--

Surface marker data are given in percent. Abbreviations: R, reference; Y, year of publication; CY, cell yield [×10^5^/ml lipoaspirate]; --, data not provided.

(a), CD34^+^/CD31^-^/CD45^-^ cells

(b), CD34^+^/CD31^-^ cells

(c), CD34^-^/CD31^+^ cells

(d), CD45^-^/CD73^+^/CD90^+^ cells

(e), CD34^+^/CD90^+^/CD45^-^/CD146^-^ cells

(f), CD34^-^/CD45^+^/CD14^+^ cells

(g), CD34^+^/CD90^-^/CD31^-^/CD45^-^/CD105^-^/CD146^+^ cells

(h), CD34^+^/CD90^+^/CD31^+^/CD45^-^/CD105^-^/CD146^+^ cells

(i), CD34^+^/CD31^+^ cells

(j) CD34^+^/CD90^+^/CD31^-^/CD45^-^/CD105^-^/CD146^-^ cells

(k), CD34^+^/CD90^+^/CD31^+^/CD45^-^/CD105^-^/CD146^+^ cells

(l), CD34^-^/ CD31^-^/CD45^+^/CD105^-^/CD14^-^/CD15^+^ cells

(m), CD90^+^/CD105^+^/CD31^-^ cells.

**Table 7:** Relative amount of ADRCs expressing certain surface markers as reported in studies describing non-enzymatic methods for isolating ADRCs.

R	Y	CY	CD13	CD29	CD34	CD44	CD73	CD90	CD31	CD45
[39]	2015	**0.07**	--	--	--	--	--	--	--	--
[43]	2014	**0.11**	--	--	--	--	--	--	--	--
[48]	2014	**0.12**	--	--	--	--	60^(a)^	^(a)^	--	^(a)^
[48]	2014	**0.23**	--	--	--	--	60^(a)^	^(a)^	--	^(a)^
[58]	2013	**0.25**	--	48	24	5	9	23	--	82
[39]	2015	**0.30**	--	--	--	--	--	--	--	--
[64]	2014	**1.25**	--	--	--	--	--	--	--	--
[56]	2014	**1.39**	--	--	--	--	--	--	--	--
[65]	2009	**2.40**	--	--	--	--	--	--	--	--
[42]	2015	**4.44**	--	--	25^(a)^	--	--	--	--	8

Surface marker data are given in percent. Abbreviations: R, reference; Y, year of publication; CY, cell yield [×10^5^/ml lipoaspirate]; --, data not provided.

(a) CD45^-^/CD73^+^/CD90^+^ cells

(b), CD34^+^/CD31^-^/CD45^-^ cells.

Most importantly, the data summarized in Tables 6 and 7 do not show any correlation between the cell yield and any single surface marker or any combination of surface markers, respectively. Neither do these data allow any differentiation between reported enzymatic and non-enzymatic methods for isolating ADRCs from human adipose tissue. Rather, the vast majority of reported enzymatic and non-enzymatic methods were not characterized according to the position statements published by IFATS and ISCT [73, 75].

Considering the available data summarized in Tables 6 and 7 and the general concerns about characterizing ADRCs and MSCs by surface markers outlined above it appears reasonable to hypothesize that determining surface markers of ADRCs is in principle not suitable for characterizing a method for isolating ADRCs from adipose tissue. This was the reason why no such characterization was performed in this study.

In any case, the available surface marker data do not allow to conclude that using enzymes in the process of isolating ADRCs from human adipose tissue subjects the cells to substantial manipulation. Rather, the available surface marker data suggest that using enzymes in the process of isolating ADRCs from human adipose tissue does not subject the cells to substantial manipulation.

Of importance, after processing cells with Matrase Reagent following the manufacturer’s instructions for use, the collagenase activity in the final cell suspension was not present (Fig 15). This suggests that the enzyme has only a supportive function in releasing the cells but has no presence or effect in the final cell suspension.

In summary, this study demonstrates that isolating ADRCs with the use of Matrase Reagent did not alter or induce biological characteristics, physiological functions or structural properties of the cells relevant for the intended use. Due to the high yield of viable, pluripotent cells in the final cell suspension isolated from lipoaspirate (or adipose tissue in general) with the Transpose RT system and the use of Matrase Reagent, cells are neither required to be cultured nor expanded, nor is any genetic manipulation necessary such as overexpression of embryonic genes as in the case of iPS cells.

Regarding the definition of minimal manipulation of cells, the U.S. Food and Drug Administration (FDA) has stated the following: “*Processing that does not alter the relevant biological characteristics of cells*” and “*Examples of relevant biological characteristics of cells or nonstructural tissues include differentiation and activation state*, *proliferation potential*, *and metabolic activity*” [77]. Furthermore, Transpose RT / Matrase isolation of ADRCs is performed directly at point of care, i.e., not *outside of a direct patient care setting* [78]. Recently, the FDA has approved clinical studies using Transpose RT / Matrase isolated ARDCs under Investigational Device Exemption (IDE) and not under Investigational New Drug Application (IND) [79–83].

The European Medicine Agency has defined advanced therapy medicinal products (ATMPs) as follows: “*Cells or tissues shall be considered ‘engineered’ if they fulfil at least one of the following conditions*: *1) the cells or tissues have been subject to substantial manipulation*, *so that biological characteristics*, *physiological functions or properties relevant for the intended regeneration*, *repair or replacement are achieved*. *The manipulation listed in Annex I*, *in particular*, *shall not be considered as substantial manipulations; and 2) the cells or tissues are not intended to be used for the same essential function or functions in the recipient as in the donor*” [28]. Accordingly, in report EMA/CAT/228/2013 the European Medicine Agency has classified Transpose RT / Matrase isolated ARDCs as not substantially manipulated and classified as non-ATMP [84].

## Conclusions

For the practice of medicine our findings do not support the hypothesis that ADRCs should preferentially be isolated from adipose tissue without enzyme, as proposed in some studies in the literature [37, 74]. Rather, the results of this study as well as a wealth of data from the literature (Tables 1 and 2, Figs 1 and 16) clearly show that non-enzymatic (i.e., just mechanical) isolation of ADRCs from adipose tissue may result in significantly lower cell yield (and most probably also in lower cell viability) than enzymatic isolation of ADRCs from adipose tissue. In order to obtain a comparable cell yield just by mechanical processing without enzyme, larger initial amounts of adipose tissue must be recovered, let alone the potentially higher amount of non-living cells in the final cell suspension [46]. Transpose RT / Matrase isolated ADRCs are—upon the respective induction—capable to differentiate into all three germ layers without prior genetic manipulation. In line with earlier recommendations in the literature [46] characterization of ADRCs for clinical use should include analysis of nucleated cell count, nucleated cells per milliliter of tissue processed, cellular viability, level of residual enzymatic activity and data from CFU-F assay. On the other hand, analysis of surface markers of ADRCs should no longer be considered suitable for characterizing a method for isolating ADRCs from adipose tissue.
